# Designing a novel vaccine against COVID-19 based on spike SARS-Cov-2 notable mutations using immunoinformatics approaches

**DOI:** 10.1371/journal.pone.0334662

**Published:** 2026-02-26

**Authors:** Somayyeh Rahimnahal, Shahnaz Yousefizadeh, Yahya Mohammadi

**Affiliations:** 1 Department of Animal Science, Faculty of Agriculture, Ilam University, Ilam, Iran; 2 Department of clinical and Laboratory Sciences, Faculty of Veterinary Sciences, Ilam University, Ilam, Iran; Central Laboratory for Evaluation of Veterinary Biologics, Agricultural Research Center, EGYPT

## Abstract

The rapid emergence of SARS-CoV-2 variants with spike protein mutations undermines the effectiveness of current vaccines, necessitating innovative strategies to ensure broad and lasting immunity. This study leverages an immunoinformatics approach to design two multi-epitope vaccine constructs Cov19-B (649 amino acids, 74 kDa) and Cov19-T (465 amino acids, 48 kDa) specifically targeting mutations in the spike protein observed in the Alpha, Beta, Gamma, and Omicron variants. Using sequence data retrieved from NCBI, GISAID, and UniProt, we predicted a range of epitopes, including linear B-cell, cytotoxic T lymphocyte (CTL), helper T lymphocyte (HTL), and IFN-gamma-inducing epitopes, selected for their high antigenicity, solubility, non-allergenicity, and non-toxicity. These epitopes provide extensive global population coverage: 76.83% for MHC I, 87.43% for MHC II, and 93.8% for combined epitopes. The constructs were enhanced with adjuvants—Human Beta-defensin 3, PADRE, and 50S ribosomal protein L7/L12—and connected with AAY, GPGPG, EAAAK, and KK linkers to optimize structural stability and immune activation. Codon-optimized has done using GenSmart™, and structurally stabilized via disulfide engineering (Disulfide by Design 2). Computational analyses, including molecular docking and dynamics simulations (assessing RMSD, RMSF, gyration, and MMPBSA), validated stable binding interactions with human neutralizing antibodies. Immune response simulations conducted via C-IMMSIM further confirmed the constructs’ capacity to trigger robust humoral and cellular immunity. To enable practical application, codon optimization was performed for efficient expression in prokaryotic systems. This study highlights the vital role of continuous genomic surveillance in tracking SARS-CoV-2 evolution and informs the development of next-generation vaccines. However, the study is limited to computational predictions, requiring experimental validation to confirm efficacy.

## Introduction

The COVID-19 pandemic, originating in Wuhan, China, in December 2019, caused severe symptoms including fever, coughing, and dyspnea [1,2]. The COVID-19 pandemic, originating in Wuhan, China, in December 2019, caused severe symptoms including fever, coughing, and dyspnea. Identified as a novel β-coronavirus (SARS-CoV-2) with sequence similarity to bat coronaviruses and MERS-CoV, it prompted global research into its characteristics, transmission, and therapies [3–6]. Its rapid spread highlighted the need for international action. Coronaviruses, including SARS (2002), MERS (2013), and COVID-19, belong to the Coronaviridae family, with SARS-CoV-2 possessing a large genomic RNA (26–32 kb) [4,6–11]. The spike glycoprotein (S-protein) binds to the host’s ACE2 receptor, primarily in lung alveolar cells, facilitating viral entry, replication, and transmission [12–15]. This process involves the release of the viral genome into the host cell, producing structural (spike, envelope, membrane, nucleocapsid) and nonstructural proteins (Replicase 1ab), enhancing viral infectivity [13,16].

The SARS-CoV-2 Spike (S) protein, comprising S1 and S2 subunits, is a key target for vaccines and therapeutics. The S1 subunit’s receptor-binding domain (RBD) facilitates viral entry by binding host cell receptors, while the S2 subunit mediates membrane fusion. Targeting the S protein with neutralizing antibodies or antiviral drugs, particularly against the S2 subunit, can prevent viral entry and reduce infection severity [17–19]. Monoclonal antibodies, such as CR3022, and plasma exchange from infected patients show therapeutic promise, though further studies are needed to confirm efficacy and safety [20]. Various COVID-19 vaccine candidates, including subunit, DNA, mRNA, and live attenuated or inactivated vaccines, aim to enhance safety, efficacy, and scalability to curb the pandemic.Inactivated SARS-CoV-2 vaccines, despite safety concerns like insufficient inactivation and vaccine-associated respiratory disease, have proven effective in clinical trials and are tightly regulated in approved countries [17]. Further research is needed to enhance their safety and efficacy. DNA vaccines, effective against MERS-CoV, use plasmids to produce S protein, activating both humoral and cellular immunity [21,22].

Subunit vaccines targeting recombinant S protein have been successful for SARS and MERS. However, DNA and mRNA vaccines may have limited protective effects due to dysregulated immune responses [23–26].

Multi-epitope vaccines, incorporating diverse antigenic components, offer a safer, more flexible solution, targeting multiple viral strains and generating robust immunity. [27–30]. Therefore, it becomes crucial to anticipate how immunogenic epitopes would interact with MHC. By leveraging the antigenic and immunogenic features of the Spike (S) protein, this methodology aims to maximize the vaccine’s efficacy and specificity while minimizing potential side effects. Therefore, the most important antigenic candidate for fighting SARS-CoV-2 is the glycoprotein S. Antiviral medications have been utilized as therapeutic approaches to reduce the viral burden or inhibit the spread of SARS-CoV-2 [31].

A novel betacoronavirus has been identified as the causative agent of severe acute respiratory syndrome (SARS) as reported by the Chinese Center for Disease Control (China CDC) on 19th December 2019 [1], which has since infected or killed a large number of people. Although multiple vaccines, such as Pfizer BNT162b2, AstraZeneca‘s AZD1222, Moderna’s mRNA-1273, and Janssen‘s Ad26.COV2.S were produced to combat COVID-19 during this period [32–35], highlight the need for broader-spectrum vaccines, including those targeting emerging variants [36]. In this way, it is ready to deal with the latest SARS mutants worldwide.

The ongoing SARS-CoV-2 pandemic, driven by variants like Delta and Omicron, underscores the need for vaccines that provide broad-spectrum protection [37]. The immunopathogenesis of COVID-19 is characterized by dysregulated innate and adaptive immune responses, initiated by Toll-like receptors (TLRs) such as TLR3, TLR4, and TLR7, which recognize viral components and trigger cytokine production (IL-6, TNF-α) [38]. In severe cases, TLR-driven hyperactivation leads to cytokine storms, marked by excessive IL-6 and IFN-γ, contributing to acute respiratory distress and multi-organ failure [39,40]. Adaptive immunity, involving cytotoxic T-lymphocytes (CTLs) and B-cells, is critical for viral clearance but often impaired in severe disease [41]. Current therapeutic measures, including antivirals (remdesivir), monoclonal antibodies (bamlanivimab), and immunomodulators (dexamethasone), offer partial efficacy but are limited against emerging variants [42]. Multi-epitope vaccines targeting conserved epitopes across SARS-CoV-2 proteins can address these challenges by eliciting robust humoral and cellular immunity [37]. Immunoinformatics approaches, using tools like IEDB, NetCTL, and ABCpred, enable precise epitope selection and construct design, as demonstrated in vaccines for SARS-CoV-2 and other pathogens [43,44].

The rapid spread of SARS-CoV-2 and its variants (Delta, Omicron) underscores the need for broadly protective vaccines targeting conserved epitopes to ensure durable immunity [45]. Multi-epitope vaccines, combining cytotoxic T-lymphocyte (CTL), helper T-lymphocyte (HTL), and B-cell epitopes, have emerged as a promising strategy to elicit robust humoral and cellular responses against pathogens like SARS-CoV-2, Yersinia pestis, Pseudomonas aeruginosa, and Mycobacterium tuberculosis [44,46,47]. Immunoinformatics approaches, leveraging tools such as IEDB, NetCTL, Propred, and BepiPred, enable precise epitope selection and construct design, as demonstrated in vaccines for filariasis and other infectious diseases [43]. Recent studies have also incorporated adjuvants like flagellin, CpG, and ribosomal proteins, alongside linkers (AAY, GPGPG) and disulfide engineering, to enhance immunogenicity and stability [44,47].

The rapid evolution of SARS-CoV-2, driven by mutations in its genome, particularly in the spike (S) protein, has led to the emergence of variants such as Delta and Omicron, which exhibit enhanced transmissibility and immune evasion, compromising the efficacy of existing vaccines [1,2]. These mutations, often occurring in the receptor-binding domain (RBD), alter antigenicity, reducing neutralizing antibody effectiveness and necessitating adaptive vaccine strategies [3]. Identifying and characterizing these mutations is critical for developing multi-epitope vaccines that target conserved epitopes across multiple viral proteins, ensuring broad-spectrum protection against diverse strains [4]. In this study, we designed two multi-epitope vaccine constructs, Cov19-B (B-cell-focused) and Cov19-T (T-cell-focused), using β-defensin and PADRE adjuvants, EAAAK and GPGPG linkers, and disulfide engineering to target SARS-CoV-2 variants, building on methodologies validated across multiple pathogens [48].

The availability of massive sequence data from pathogens, as well as the improvement of computational prediction methods, has substantially aided the identification of possible immunogenic epitopes in pathogen proteins in the postgenomic era [49]. This can help with the development of vaccine against certain infections. Recent studies have predicted viable vaccine candidates for viral infections using immunoinformatics approaches [50].

## Materials and methods

**Fig 1:**
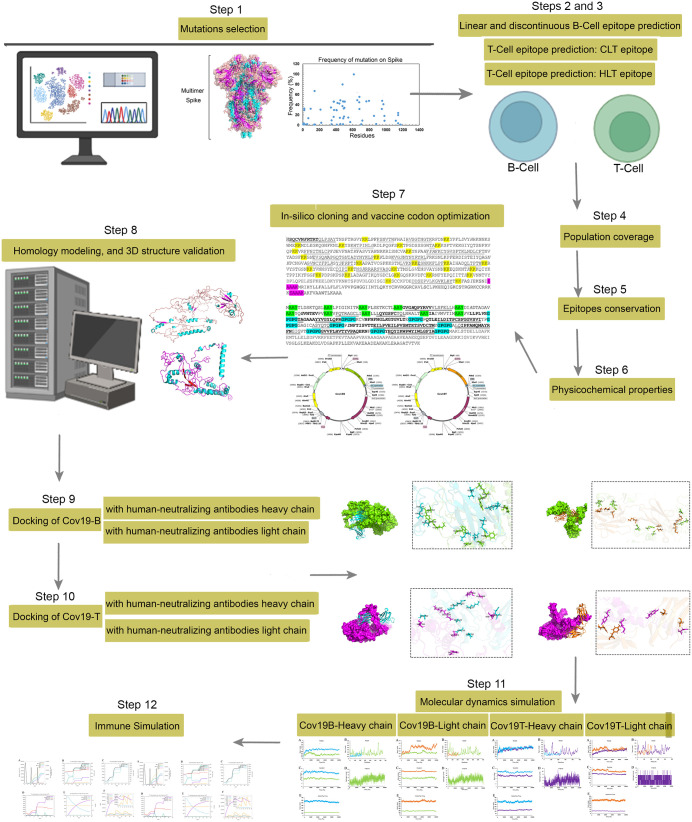
All performed steps of this study.

All steps related to this study are shown in Fig 1.

### Mutations selection

Genomic data from approximately 12 million SARS-CoV-2 sequences, deposited in the GISAID database (https://www.epicov.org) from the start of the pandemic to January 8, 2022, were retrieved. A custom script was used to extract each strain’s sequence from FASTA format files. For each viral strain, nucleotide and amino acid variations were identified through a multi-step process. The SARS-CoV-2 reference genome was indexed using bwa-build, and viral sequences were mapped to this index with bwa-mem [51]. The resulting SAM files were converted to BAM files using Samtools view v1.9, then sorted and indexed with Samtools sort and Samtools index v1.9, respectively, applying default settings. Variant calling was conducted using bcftools call v1.9 [52]. An in-house script was employed to calculate the frequencies of nucleotide and amino acid mutations within each gene.

### Linear and discontinuous B-Cell epitope prediction

Linear B-cell epitopes were predicted using the IEDB server (http://tools.iedb.org/bcell/). This server employs an approach that evaluates key polypeptide chain characteristics to determine antigenicity, utilizing amino acid scales and Hidden Markov Models (HMMs) to analyze sequence properties and identify linear B-cell epitopes.

Discontinuous B-cell epitopes were identified using the ElliPro server (http://tools.iedb.org/ellipro/). ElliPro predicts these epitopes based on the three-dimensional structure of a protein antigen, calculating a score derived from the average Protrusion Index (PI) of epitope residues. It uses the PI to detect discontinuous B-cell epitopes and clusters them based on the distance (in angstroms) between the residues’ centers of mass.

### CLT epitope prediction

The first step in designing multi-epitope vaccines using bioinformatics is to identify the immunoprotective properties of epitopes. T-cell epitopes, presented by MHC molecules, are linear and typically comprise 9 to –20 amino acid residues, enabling accurate modeling of ligand–T-cell interactions. The binding of MHC molecules to antigenic peptides is a crucial step in presenting them to T-cell receptors. In this study, we employed Artificial Neural Networks to predict T-cell epitopes with high MHC class I binding affinity, utilizing the NetMHCpan4.1 server (http://www.cbs.dtu.dk/services/NetMHCpan/). This tool predicts MHC class I binding with an accuracy of 90–95%.

### HLT epitope prediction

To predict interactions between helper T-cell (HTL) epitope peptides and MHC class II molecules, we utilized the NetMHCIIpan 4.0 server (http://www.cbs.dtu.dk/services/NetMHCIIpan/). Thresholds of 2% and 10% were set to identify strong and weak binders, respectively, based on human alleles. This method was designed to assess the binding affinity of T-cell epitope peptides to MHC class II molecules.

### Investigation of coverage in population

The IEDB population coverage server (https://tools.iedb.org/population/) was used to evaluate whether the selected vaccine epitopes provided effective coverage across the global population. Considering the global scope of the SARS-CoV-2 pandemic, the analysis included diverse regions worldwide. The assessment was performed using default settings, testing coverage for both HLA classes. This comprehensive approach sought to ensure inclusivity across varied populations.

### Epitopes conservation analysis

The IEDB conservancy analysis tool was utilized, so that epitopes could be assessed for conservation at different lengths. This analysis aimed to provide insights into how well the selected epitopes were preserved across related sequences, contributing to a better understanding of their potential effectiveness in diverse contexts.

### Physicochemical properties of the vaccine construct

The antigenicity of the vaccine design was confirmed using VaxiJen v2.0109 with a threshold value of 0.4. Whole-protein antigenicity prediction models were derived from viral databases. The server’s models demonstrated robust performance in both validations, achieving prediction accuracies within the range of 70% to 89%. To assess the vaccine’s allergenicity, the AllerTOP server was employed. The vaccine’s allergenicity was further verified using the AllergenFP server (https://ddg-pharmfac.net/AllergenFP/). The ExPASy ProtParam server was utilized to evaluate various physicochemical parameters of the vaccine. Additionally, SignalP4.1 (https://www.cbs.dtu.dk/services/SignalP/) and TMHMM server v2.0 (https://www.cbs.dtu.dk/services/TMHMM/) were employed to check for the presence of signal peptides and transmembrane helices in the vaccine design. These comprehensive analyses contribute to a thorough understanding of the vaccine’s properties and potential efficacy.

### *In-silico* cloning and vaccine codon optimization

To construct effective vaccine components, antigenic epitopes were fused using specific peptide linkers. Three distinct constructions were created for each linear B lymphocyte (LBL), cytotoxic T lymphocyte (CTL), and helper T lymphocyte (HTL). For optimizing codons, the GenSmart™ Codon Optimization available at https://www.genscript.com/tools/gensmart-codon-optimization was employed. The Codon Adaptation for estimating protein expression, were set at 1.0 and 30–70%, respectively. The goal of codon optimization was to maximize expression in strain K-12 *Escherichia coli*. The rho-independent transcription terminators and bacterial ribosome binding sites were all avoided by setting the parameters. To identify *NdeI* and *XhoI* restriction enzyme sites, the SnapGene tool was used.

### Homology modeling, and 3D structure validation

The 3D structure of the construct was modeled using I-TASSER (Iterative Threading ASSEmbly Refinement), as developed by I-TASSER.

To improve the accuracy and quality of the 3D model, refinement was conducted using the ModRefiner tool (https://zhanglab.ccmb.med.umich.edu/ModRefiner). This process enhances the precision and reliability of the 3D structure predictions, ensuring an accurate representation of the vaccine construct’s spatial configuration. The stereochemical quality of the protein structure was evaluated using PROCHECK (http://services.mbi.ucla.edu/PROCHECK), which analyzes parameters such as bond lengths, bond angles, and dihedral angles to assess the model’s quality. The SAVES server (http://services.mbi.ucla.edu/SAVES) was employed to calculate the Z-Score for the 3D structures of Cov19-B and Cov19-T. SAVES integrates multiple validation programs to evaluate stereochemical properties, side-chain parameters, and other structural features, ensuring the reliability of the modeled structures. The Ramachandran plot, generated via the MolProbity server (http://molprobity.biochem.duke.edu/), was used to visualize the distribution of phi and psi dihedral angles, facilitating the identification of potential issues in the backbone conformation of the modeled structures.

### Disulfide engineering of Cov19-B and Cov19-T

To enhance the structural stability of the vaccine constructs, disulfide bond engineering was performed using the Disulfide by Design 2.0 (DbD2) server. The refined 3D models of Cov19-B and Cov19-T were analyzed to identify residue pairs suitable for disulfide bond formation. Criteria for selection included: (i) spatial proximity of residue pairs (Cα-Cα distance ~4–6 Å), (ii) favorable bond geometry (χ3 torsion angle ~±90°), and (iii) minimal disruption to epitope antigenicity. Non-cysteine residues meeting these criteria were mutated to cysteines to introduce disulfide bonds. The number of disulfide bonds introduced was optimized to balance stability and flexibility, ensuring no interference with MHC binding sites.

### Docking of vaccine construct with antibody

The 3D structure of the human-neutralizing antibody (PDB ID: 7BWJ) was obtained from the RCSB server. The 3D structure of 7BWJ was optimized using PyMOL v2.3.4, and hydrogen peroxide molecules were removed from the PDB structures of the vaccine constructs and 7BWJ. To identify interaction zones, the prepared 3D structures of Cov19-B, Cov19-T, and 7BWJ were submitted to the HDOCK server after preprocessing with Chimera. Complexes exhibiting the lowest mean Root Mean Square Deviation (RMSD) and intermolecular binding energy between the vaccine constructs and the heavy and light chains of 7BWJ were selected based on HADDOCK results. LigPlot+ was utilized to evaluate hydrogen bond formation, providing insights into the hydrogen bond interactions within the complexes. This rigorous approach aimed to elucidate potential binding and interaction patterns between the 7BWJ antibody and the Cov19-B/T constructs.

### Simulation of complexes by MD

Molecular dynamics simulations of the vaccine-antibody complexes were performed using GROMACS v4.6.5 with the CHARMM36 all-atom force field. The protein–protein complexes were solvated with water molecules, and the system was neutralized. Energy minimization was conducted to stabilize the system’s configuration. Equilibration was carried out under NVT (constant Number of particles, Volume, and Temperature) and NPT (constant Number of particles, Pressure, and Temperature) conditions at 300 K, 1 bar, and restraint forces of 1000 kJ/mol. Production simulations were run for 100,000 ps (100 ns). All bonds were constrained using the LINCS algorithm to maintain stability and prevent unrealistic bond length variations. These procedures collectively enabled the simulation of the dynamic behavior of the vaccine-antibody complexes over an extended period. The use of GROMACS and the CHARMM36 force field provided a robust framework for precise and detailed molecular dynamics simulations.

### Estimation of binding-free energies

The MMPBSA method calculates binding free energies in molecular dynamics simulations, offering critical insights into the energetics of protein–protein or protein–ligand interactions. The MMPBSApy module from AMBER16 was utilized to compute Molecular Mechanics/Poisson-Boltzmann Surface Area (MMPBSA) binding free energies for the receptors and the multi-epitope peptide vaccine construct. Input files for the complex, receptor, and vaccine construct were generated using the ante-MMPBSA.py module. From the simulation trajectories, 100 frames were extracted to analyze the energy differences between solvated and unsolvated states. Binding free energies were accurately determined by comparing two distinct conformations and assessing the contributions of key residues to the binding energetics. To calculate the free binding energy (ΔGbind, solv) of the anticipated complex, three equations (Eqn 1, Eqn 2, Eqn 3) were utilized. These equations involve terms related to the solvation-free energy, van der Waals interactions, electrostatic interactions, and entropy contributions [53,54]:


$${\Delta \text{G}}_{\text{bind},\text{solv }}={\Delta \text{G}}_{\text{bind},\text{vaccum}}+\;{\Delta \text{G}}_{\text{Solv},\text{complex}}-{\Delta \text{G}}_{\text{solv},\text{ligand}}-{\Delta \text{G}}_{\text{solv},\text{complex}}$$ (1)

$${\Delta \text{G}}_{\text{solv}}={\Delta \text{G}}_{\text{electrostatic}}(\in_{\text{8}\text{0}-\text{1}})+{\Delta \text{G}}_{\text{hydrophobic}}\;$$ (2)

$${\Delta \text{G}}_{\text{vaccum}}={\Delta \text{E}}_{\text{molecular mechanics}}-\text{T}.\;{\Delta \text{G}}_{\text{normal mode analysis}}$$ (3)

The net free binding energy was broken down into each residue in order to emphasize the stable and interacting residues.

### Immune simulation

The immunogenicity and immune response profile induced by the Cov vaccine were evaluated using the C-ImmSim server, which employs machine learning techniques to predict immune responses. A minimum interval of four weeks was set between the first and second doses. The simulation spanned 1050-time steps, with each time step corresponding to approximately eight hours. Three injections were administered at four-week intervals, corresponding to time steps 1, 84, and 168.

### Ethics statement

This study does not contain any human/animal samples. All authors agree on the Ethical Approval and Consent to participate.

## Results

### Sequence retrieve

The spike sequence of SARS-Cov-2, with protein ID of P0DTC2, was selected from UniProt based on previous studies.

### Selection of mutation

**Fig 2:**
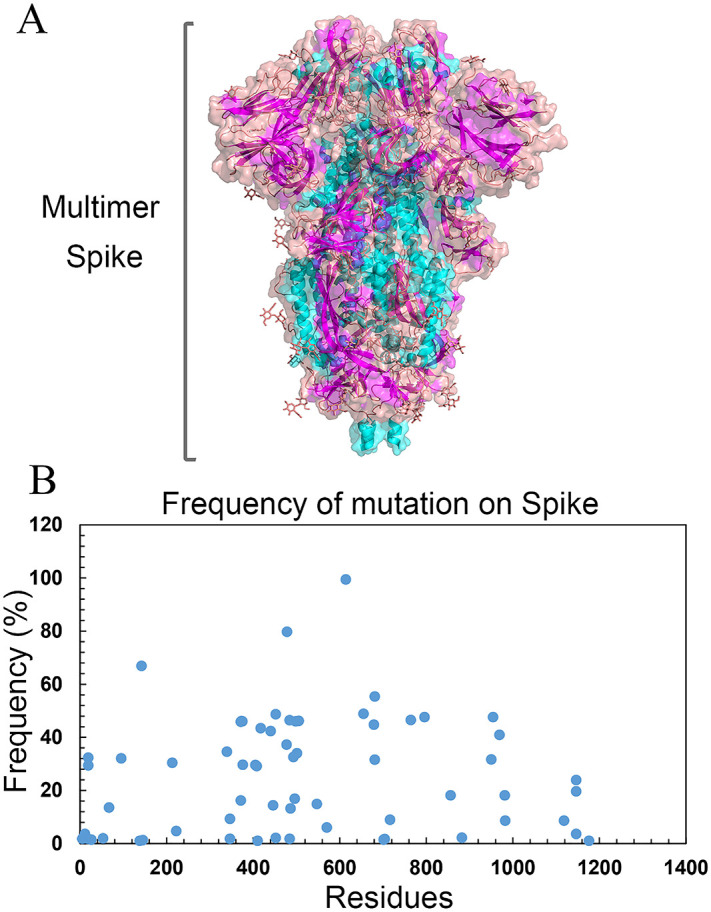
(A) The multimeric (tetramer) structure of the SARS-CoV-2 Spike protein, derived from the PDB entry 6VXX (RCSB Protein Data Bank), visualized using PyMOL software. The structure highlights the trimeric arrangement of the Spike glycoprotein, with distinct domains including the receptor-binding domain (RBD) and S2 subunit, critical for viral entry and immune recognition. Different chains are color-coded to illustrate the quaternary organization, providing a foundation for identifying conserved regions targeted in the design of the multi-epitope vaccine constructs [55] and visualized by PyMol. (B) Scatter plot depicting the frequency of mutations across the Spike protein sequence (residues 1 to 1400), based on genomic data analysis of SARS-CoV-2 variants. The x-axis represents residue positions, while the y-axis indicates mutation frequency (in percentage). High mutation frequencies, particularly in the RBD (residues ~330–530), underscore the virus’s immune evasion potential, guiding the selection of conserved epitopes for vaccine design to ensure broad-spectrum protection against evolving variants.

All mutations with frequencies higher than 1% were extracted from the spike sequence and used for further study (Table 1). A multimer/tetramer structure of the Spike (RCSB code: 6VXX) is shown in Fig 2A. A plot of the frequency of mutations across the spike sequence is shown in Fig 2B.

**Table 1:** Mutations and frequency related to them.

Position	Mutation	Freq %
23403-A > G	Asp614Gly	99.44165802
22995-C > A	Thr478Lys	79.72207538
21987-G > A	Gly142Asp	66.94911996
23604-C > A	Pro681His	55.35651463
23525-C > T	His655Tyr	48.86557158
22917-T > G	Leu452Arg	48.73775845
23948-G > T	Asp796Tyr	47.66834967
24424-A > T	Gln954His	47.61932713
23854-C > A	Asn764Lys	46.54524236
23013-A > C	Glu484Ala	46.43867722
23075-T > C	Tyr505His	46.19597669
22679-T > C	Ser373Pro	46.11882008
23055-A > G	Gln498Arg	46.0646704
22686-C > T	Ser375Phe	46.00533655
22674-C > T	Ser371Phe	45.84232707
23599-T > G	Asn679Lys	44.75927153
22813-G > T	Lys417Asn	43.44176343
22882-T > G	Asn440Lys	42.38818375
24469-T > A	Asn969Lys	40.92896411
22992-G > A	Ser477Asn	37.32094758
22578-G > A	Gly339Asp	34.642319
23063-A > T	Asn501Tyr	34.06625454
23040-A > G	Gln493Arg	32.64114954
21618-C > G	Thr19Arg	32.39799839
21846-C > T	Thr95Ile	32.10654429
24410-G > A	Asp950Asn	31.72864897
23604-C > G	Pro681Arg	31.62427746
22200-T > G	Val213Gly	30.40932706
22688-A > G	Thr376Ala	29.67216119
22775-G > A	Asp405Asn	29.63616076
21618-C > T	Thr19Ile	29.44195645
22786-A > C	Arg408Ser	29.17804051
25000-C > G	Asp1146Glu	23.94850787
25000-C > A	Asp1146Glu	19.68103396
24503-C > T	Leu981Phe	18.16519759
24130-C > A	Asn856Lys	18.1411527
23048-G > A	Gly496Ser	16.89613427
22673-T > C	Ser371Pro	16.23163694
23202-C > A	Thr547Lys	14.95381764
22898-G > A	Gly446Ser	14.4036761
21762-C > T	Ala67Val	13.59290888
23018-T > G	Phe486Val	13.25675081
22599-G > A	Arg346Lys	9.365489779
23709-C > T	Thr716Ile	8.96303551
24914-G > C	Asp1118His	8.646328087
24506-T > G	Ser982Ala	8.636724078
23271-C > A	Ala570Asp	6.09689089
22227-C > T	Ala222Val	4.731204307
25000-C > T	Asp1146Asp	3.66984066
21595-C > T	Val11Val	3.65333019
24208-C > T	Ile882Ile	2.241413709
22917-T > A	Leu452Gln	2.172014521
21721-C > A	Asp53Glu	1.9665473
21614-C > T	Leu18Phe	1.829698906
23012-G > T	Glu484*	1.789557279
22599-G > C	Arg346Thr	1.781074898
21575-C > T	Leu5Phe	1.776573914
23673-C > A	Ser704*	1.736716901
23664-C > T	Ala701Val	1.509961627
21638-C > T	Pro26Ser	1.473789653
21995-T > C	Tyr145His	1.326935447
25088-G > T	Val1176Phe	1.093193526
22792-C > G	Ile410Met	1.081920843
21974-G > T	Asp138Tyr	1.057996786

### Prediction of B-cell epitopes: Linear

Twenty-six linear B-cell epitopes were chosen for their mutations, aiming to stimulate humoral immunity through the vaccine. Predicted B-cell epitopes, and subsequent evaluation of antigenicity was carried out (Table 2). Also, all mutations related to each epitope are shown in Table 2. Mutations were applied to the final construct.

**Table 2:** B-cell epitope selected alongside their mutation. Overlapped sequence/epitope wit CTL epitopes and HTL epitopes are shown as underlined.

No.	Start	End	Peptide	Length	Mutation/s
1	13	37	SQCVNLTTRTQLPPAYTNSFTRGVY	25	Leu18Phe, Pro26Ser, Thr19Arg, Thr19Ile,
2	59	81	FSNVTWFHAIHASGTNGTKRFDN	23	Ala67Val
3	138	154	DPFLGVYYHKNNKSWME	17	Asp138Tyr, Gly142Asp, Tyr145His
4	177	189	MDLEGKQGNFKNL	13	-
5	206	221	KHTPINLVRDLPQGFS	16	Val213Gly
6	250	260	TPGDSSSGWTA	11	-
7	304	322	KSFTVEKGIYQTSNFRVQP	19	-
8	329	363	FPNITNLCPFGEVFNATRFASVYAWNRKRISNCVA	35	Gly339Asp, Arg346Lys, Arg346Thr
9	369	393	YNSASFSTFKCYGVSPTKLNDLCFT	25	Ser371Pro, Ser371Phe, Ser373Pro, Ser375Phe, Thr376Ala
10	404	426	GDEVRQIAPGQTGKIADYNYKLP	23	Asp405Asn, Arg408Ser, Ile410Met, Lys417Asn
11	440	501	NLDSKVGGNYNYLYRLFRKSNLKPFERDISTEIYQAGSTPCNGVEGFNCYFPLQSYGFQPTN	62	Asn440Lys, Gly446Ser, Leu452Gln, Leu452Arg, Ser477Asn, Thr478Lys, Phe486Val, Glu484Ala, Gln493Arg, Gly496Ser, Gln498Arg, Asn501Tyr
12	516	536	ELLHAPATVCGPKKSTNLVKN	21	-
13	555	562	SNKKFLPF	8	-
14	616	632	NCTEVPVAIHADQLTPT	17	-
15	634	644	RVYSTGSNVFQ	11	-
16	656	666	HVNNSYECDIPI	11	His655Tyr
17	672	690	ASYQTQTNSPRRARSVASQ	19	Pro681His
18	695	710	YTMSLGAENSVAYSNN	16	Ala701Val
19	773	779	EQDKNTN	7	
20	786	800	KQIYKTPPIKDFGGF	15	Asp796Tyr
21	807	814	PDPSKPSK	8	-
22	828	842	LADAGFIKQYGDCLG	15	-
23	1035	1043	GQSKRVDFC	9	-
24	1107	1118	RNFYEPQIITTD	12	Asp1118His
25	1133	1172	VNNTVYDPLQPELDSFKEELDKYFKNHTSPDVDLGDISGI	40	Asp1146Glu
26	1252	1267	SCCKFDEDDSEPVLKG	16	-

### Prediction of the cytotoxic T lymphocyte epitope

Given the importance of fostering cellular immunity, an evaluation of the S protein was carried out. As shown in Table 3, the 73 CTL epitopes were chosen for their antigenicity and binding affinity. All mutations related to each epitope are shown in Table 3. Mutations were incorporated into the final construct.

**Table 3:** CTL epitope selected alongside their mutation. Overlapped sequence/epitope with B-cell epitopes and HTL epitopes are shown as underlined. Repetitive CTL epitopes are shown in bold characters.

Pos.	Allele	peptide	1-log50k(aff)	affinity(nM)	Vaxigen	Mutation/s
15	HLA-B1402	VN **LRTRTQL**	0.297	2009.70	1.4448	Leu18Phe, Pro26Ser, Thr19Arg, Thr19Ile,
17	HLA-B7301	**LRTRTQLPP**	0.160	8881.44	1.4299	Leu18Phe, Pro26Ser, Thr19Arg, Thr19Ile,
18	HLA-A3001	**RTRTQLPPA**	0.934	2.05	1.2776	Leu18Phe, Pro26Ser, Thr19Arg, Thr19Ile,
19	HLA-C0702	**TRTQLPPA** Y	0.367	944.13	1.2923	Leu18Phe, Pro26Ser, Thr19Arg, Thr19Ile,
55	HLA-B8301, HLA-B5401, HLA-B5301, HLA-B5101, HLA-A6823, HLA-A3215,	LPFFSNVTW	0.342	1230.56	1.0808	-
68	HLA-A3001, HLA-A2603	HVSGTNGTK	0.616	63.75	1.0956	-
92	HLA-C0303, HLA-C0802, HLA-C1203	FASTEKSNI	0.589	85.04	1.0137	Thr95Ile
109	HLA-B0802, HLA-B1402, HLA-C0802, HLA-A0219	TLDSKTQSL	0.142	10745.41	1.0685	-
201	HLA-C1203, HLA-C0802, HLA-C0303	Y**SKHTPINL**	0.697	26.57	1.0547	-
202	HLA-C0602	**SKHTPINL** V	0.385	773.28	1.1051	-
229	HLA-B8301	LPIGINITR	0.331	1384.58	1.8215	-
262	HLA-C1402, HLA-C0702, HLA-A2603, HLA-A6601, HLA-A3301, HLA-A3207	YYVGYLQPR	0.714	22.05	1.4692	-
292	HLA-A0216, HLA-A0250	PLSETKCTL	0.612	66.24	1.0147	-
324	HLA-C1203, HLA-C0702, HLA-C0602, HLA-C0701, HLA-B1402	V**RFPNITNL**	0.579	94.62	1.1141	-
325	HLA-A2403	**RFPNITNLC**	0.682	31.20	1.2172	-
326	HLA-B5301	**FPNITNLC** P	0.661	39.11	1.6218	-
375	HLA-A3001	K **CYGVSPTK**	0.721	20.53	1.4199	-
376	HLA-C0401, HLA-A2403	**CYGVSPTK** L	0.206	5395.54	1.4263	-
384	HLA-C0501	**L**NDLCFTNV	0.463	334.57	2.0179	-
403	HLA-A2603	EV **RQIAPGQ**	0.342	1237.09	1.1205	-
405	HLA-B4801	**RQIAPGQ** TG	0.273	2604.47	1.7890	-
414	HLA-A3201, HLA-A0212, HLA-B4201, HLA-A0201, HLA-A0202, HLA-C1502, HLA-A0250	K **IADYNYKL**	0.596	79.51	1.6639	-
415	HLA-C0802, HLA-C0501	**IADYNYKL**P	0.234	3977.74	1.1012	-
441	HLA-A3101	K **VGGNYNYL**	0.807	8.09	1.5212	-
442	HLA-A3002, HLA-A8001, HLA-A8001	**VGGNYNYLY**	0.692	27.98	1.7683	-
443	HLA-A3101	**GGNYNYLYR**	0.662	38.80	1.6845	-
444	HLA-B7301, HLA-B4013, HLA-A3215, HLA-B4801	**GNYNYLYR** L	0.185	6736.21	1.5305	-
482	HLA-C0401	GFNCYFPLQ	0.227	4290.90	1.2939	Glu484Ala
492	HLA-A6601, HLA-A6901, HLA-C1203	YGFQPTN **GV**	0.233	4000.75	1.0509	Gly496Ser
503	HLA-C1203	**VGYQPYRVV**	0.571	103.28	1.4383	Tyr505His
507	HLA-A2301, HLA-A2403, HLA-A2402, HLA-C0702	**PYRVV** VLSF	0.684	30.39	1.0281	-
509	HLA-A3201, HLA-B4801	**RVV** VLSFEL	0.472	304.18	1.1918	-
511	HLA-A8001	**VV**LSFELLH	0.424	509.61	1.4090	-
530	HLA-B0802, HLA-B0803	LV **KNKCVNF**	0.133	11808.59	1.3263	-
532	HLA-B5802, HLA-B0803	**KNKCVNFNF**	0.147	10141.50	2.4991	-
535	HLA-A6802	**CVNFNF** NGL	0.707	23.74	1.7985	-
551	HLA-A2603, HLA-C1502, HLA-B0801, HLA-B0802, HLA-A2602, HLA-A6601	ESNKKFLPF	0.328	1436.35	1.0278	-
568	HLA-A2501, HLA-A6901, HLA-A6802	DIADTADAV	0.278	2481.74	1.0904	Ala570Asp
581	HLA-C0501	ILD **ITPCSF**	0.550	129.51	1.1835	-
584	HLA-A0206, HLA-A2603, HLA-A6901, HLA-A6802	**ITPCSFGGV**	0.702	25.07	1.3871	-
587	HLA-B4601, HLA-B4013, HLA-C1203	**CSFGGV** SVI	0.244	3550.53	1.2630	-
612	HLA-A0250, HLA-A0206, HLA-A0212	YQ**DVNCTEV**	0.750	14.95	1.3957	Asp614Gly
614	HLA-A0216, HLA-A0212	**DVNCTEV**PV	0.689	28.90	1.0848	Asp614Gly
625	HLA-A2602, HLA-A3207	**QLTPT**WRVY	0.543	139.66	1.2119	-
626	HLA-A6823	**LTPT**WRVYS	0.474	296.34	1.1258	-
640	HLA-C1402, HLA-C0401	VFQTRAGCL	0.545	137.52	1.7094	-
642	HLA-A0206, HLA-C0802, HLA-B4013	FQTRAGCLI	0.71	23.04	1.7332	-
678	HLA-B0802, HLA-B0801, HLA-B1402, HLA-C1502	N **SPRRARSV**	0.137	11407.84	1.2335	Pro681Arg
679	HLA-B1402, HLA-B7301	**SPRRARSVA**	0.335	1329.94	1.4417	Pro681Arg
680	HLA-B0803, HLA-B1402	**PRRARSVA** S	0.158	9034.48	1.1645	Pro681Arg
722	HLA-A1101, HLA-A6801	EI **LPVSMTK**	0.620	60.91	1.6842	-
724	HLA-B5401	**LPVSMTKTS**	0.508	206.02	1.5550	-
730	HLA-A0101, HLA-A8001, HLA-B5801, HLA-A3002	**KT**SVDCTMY	0.399	666.37	1.1824	-
752	HLA-A0216, HLA-A0250	LLL**QYGSFC**	0.625	57.61	1.3260	-
755	HLA-C0401, HLA-A2403	**QYGSFC** TQL	0.251	3308.58	1.2906	-
891	HLA-B4801, HLA-B1501, HLA-A0206, HLA-B4013	**LQIPFAMQM**	0.223	4479.03	1.0680	-
893	HLA-A2902, HLA-A3215, HLA-B5301, HLA-B5101, HLA-A8001, HLA-A6823, HLA-B8301	**IPFAMQMAY**	0.697	26.63	1.4278	-
894	HLA-A3301	**PFAMQMAYR**	0.590	84.41	1.3315	-
895	HLA-B0803, HLA-C0501, HLA-A2301, HLA-A2402, HLA-A2403, HLA-B0801, HLA-B0802, HLA-A2902, HLA-A3201, HLA-A3207, HLA-A3215, HLA-A6601, HLA-C1203, HLA-C1502, HLA-C0702, HLA-C0602, HLA-B5802, HLA-B5801, HLA-B8301, HLA-C0303, HLA-A6823, HLA-B4601, HLA-B4013, HLA-B5101, HLA-B5301	**FAMQMAYR** F	0.168	8082.94	1.0278	-
900	HLA-A3001	A **YRFNGIGV**	0.714	22.18	1.2995	-
901	HLA-B7301, HLA-C1203, HLA-C0701, HLA-C0702, HLA-C0602,	**YRFNGIGV**T	0.292	2118.85	1.7692	-
1056	HLA-A2902, HLA-A6823, HLA-A8001,	G **VVFLHVTY**	0.724	19.76	1.4104	-
1057	HLA-A0203, HLA-A0205, HLA-A0206, HLA-C1203, HLA-C0602, HLA-C0701, HLA-A0219, HLA-A0201	**VVFLHVTYV**	0.763	13.02	1.5122	-
1059	HLA-A0211, HLA-A0212, HLA-A0216, HLA-A0203, HLA-B0803, HLA-B5401,	**FLHVTYV** PA	0.787	10.04	1.3346	-
1204	HLA-B7301, HLA-B4013, HLA-A6823, HLA-A6601, HLA-A3207, HLA-A2603,	E **QYIKWPWY**	0.185	6770.04	1.1384	-
1205	HLA-A0205, HLA-A2301, HLA-A2402, HLA-A2403, HLA-A3207, HLA-A3215, HLA-A6823, HLA-C0702, HLA-B4013,	**QYIKWPWYI**	0.282	2367.64	1.4177	-
1208	HLA-A2402	**KWPWYIWLG**	0.536	151.97	1.0478	-
1209	HLA-B0802, HLA-A2603, HLA-A3215, HLA-A6823, HLA-B4013, HLA-B4201, HLA-B5101, HLA-B5301, HLA-C0401, HLA-B7301, HLA-B8301,	**WPWYIWLGF**	0.139	11141.67	1.4953	-
1211	HLA-B7301	**WYIWLGFIA**	0.206	5363.29	1.0356	-
1225	HLA-B5101, HLA-B4601, HLA-B5801,	**IA**IVMVTIM	0.283	2333.84	1.1339	-
1259	HLA-B8301	EP **VLKGVKL**	0.282	2365.80	1.2301	-
1261	HLA-A8001	**VLKGVKL** HY	0.478	282.69	1.2378	-

### Selection of helper T Lymphocyte Epitopes

40 HTL epitopes were investigated with an emphasis on finding strong-binding and highly antigenic epitopes (Table 4). Furthermore, each selected HTL epitope’s ability to induce IFN-γ is shown in Table 4.

**Table 4:** HTL epitope selected alongside their mutation. Overlapped sequence/epitope with B-cell epitopes and CTL epitopes are shown as underlined. Repetitive HTL epitopes are shown in bold characters.

Allele	pos	peptide	1-log50k (aff)	Affinity (nM)	Vaxigen	Mutation
DRB3_0301	5	LV**LLPLVSSQCVNLT**	0.8213	6.9	1.2086	Leu5Phe, Val11Val, Leu18Phe, Thr19Arg, Thr19Ile
DRB4_0101	5	LV**LLPLVSSQCVNLT**	0.68	31.9	1.2086	Leu5Phe, Val11Val, Leu18Phe, Thr19Arg, Thr19Ile
HLA-DQA10102-DQB10501	5	LV**LLPLVSSQCVNLT**	0.8458	5.3	1.2086	Leu5Phe, Val11Val, Leu18Phe, Thr19Arg, Thr19Ile
DRB3_0301	7	**LLPLVSSQCVNLTTR**	0.8139	7.5	1.2748	Val11Val, Leu18Phe, Thr19Arg, Thr19Ile
DRB4_0101	7	**LLPLVSSQCVNLTTR**	0.6948	27.2	1.2748	Val11Val, Leu18Phe, Thr19Arg, Thr19Ile
HLA-DQA10102-DQB10501	7	**LLPLVSSQCVNLTTR**	0.7838	10.4	1.2748	Val11Val, Leu18Phe, Thr19Arg, Thr19Ile
HLA-DQA10301-DQB10301	259	**TAGAAAYYVGYLQPR**	0.7262	19.4	1.0413	-
HLA-DQA10104-DQB10503	259	**TAGAAAYYVGYLQPR**	0.489	251.9	1.0413	-
HLA-DQA10501-DQB10402	374	**FSTFKCYGVSPTKLN**	0.6827	31.0	1.0042	Ser375Phe, Thr376Ala
HLA-DQA10601-DQB10402	374	**FSTFKCYGVSPTKLN**	0.5650	110.7	1.0042	Ser375Phe, Thr376Ala
HLA-DQA10201-DQB10402	374	**FSTFKCYGVSPTKLN**	0.6471	45.5	1.0042	Ser375Phe, Thr376Ala
HLA-DQA10501-DQB10402	375	**STFKCYGVSPTKLND**	0.6642	37.80	1.2022	Ser375Phe, Thr376Ala
HLA-DQA10201-DQB10402	375	**STFKCYGVSPTKLND**	0.6132	65.7	1.2022	Ser375Phe, Thr376Ala
HLA-DQA10501-DQB10201	386	**KLNDLCFT**NVYADSF	0.59	86.90	1.1791	-
HLA-DQA10501-DQB10402	487	NCYFPLQSYGFQPTN	0.6985	26.1	1.0385	-
HLA-DQA10501-DQB10402	502	G**VGYQPYRVV**VLSFE	0.7166	21.5	1.3049	Tyr505His
HLA-DQA10303-DQB10402	502	G**VGYQPYRVV**VLSFE	0.5204	179.3	1.3049	Tyr505His
HLA-DQA10303-DQB10402	503	**VGYQPYRVV**VLSFEL	0.4826	269.8	1.3858	Tyr505His
HLA-DQA10501-DQB10402	503	**VGYQPYRVV**VLSFEL	0.6946	27.2	1.3858	Tyr505His
HLA-DPA10103-DPB10601	504	**GYQPYRVV**VLSFELL	0.8300	6.3	1.0740	Tyr505His
DRB1_0901	537	K**CVNFNFNGLTGTGV**	0.6982	26.2	1.5826	Thr547Lys
DRB1_0901	538	**CVNFNFNGLTGTGVL**	0.7413	16.4	1.3281	Thr547Lys
DRB1_0101	539	**VNFNFNGLTGTGVLT**	0.8064	8.1	1.2439	Thr547Lys
DRB1_0901	539	**VNFNFNGLTGTGVLT**	0.7522	14.6	1.2439	Thr547Lys
DRB1_0101	540	**NFNFNGLTGTGVLT**E	0.8104	7.8	1.0516	Thr547Lys
DRB1_0901	540	**NFNFNGLTGTGVLT**E	0.7540	14.3	1.0516	Thr547Lys
DRB1_0401	754	LQYGSFCTQLNRALT	0.6512	43.6	1.0270	-
HLA-DQA10102-DQB10501	579	P**QTLEILDITPCSFG**	0.7707	12	1.5270	-
HLA-DQA10102-DQB10501	580	**QTLEILDITPCSFGG**	0.7686	12.2	1.4946	-
HLA-DQA10102-DQB10501	581	**TLEILDITPCSFGGV**	0.7659	12.6	1.6214	-
HLA-DQA10501-DQB10303	586	**DITPCSFGGV**SVITP	0.5856	88.60	1.2254	-
HLA-DQA10301-DQB10301	586	**DITPCSFGGV**SVITP	0.6071	70.1	1.2254	-
HLA-DQA10301-DQB10301	663	**DIPIGAGICASYQTQ**	0.6245	58.1	1.1088	-
HLA-DQA10501-DQB10301	663	**DIPIGAGICASYQTQ**	0.6831	30.8	1.1088	-
HLA-DQA10601-DQB10402	715	**PTNFTISVTTEILPV**	0.5586	118.6	1.1349	Thr716Ile
HLA-DQA10201-DQB10303	715	**PTNFTISVTTEILPV**	0.6474	45.4	1.1349	Thr716Ile
DRB1_0701	715	**PTNFTISVTTEILPV**	0.8083	8.0	1.1349	Thr716Ile
DRB1_0901	715	**PTNFTISVTTEILPV**	0.7523	14.6	1.1349	Thr716Ile
HLA-DQA10201-DQB10202	715	**PTNFTISVTTEILPV**	0.5697	105.1	1.1349	Thr716Ile
HLA-DQA10201-DQB10402	715	**PTNFTISVTTEILPV**	0.6237	58.7	1.1349	Thr716Ile
DRB1_0701	716	**TNFTISVTTEILPVS**	0.8056	8.2	1.1691	Thr716Ile
DRB1_0901	716	**TNFTISVTTEILPVS**	0.7245	19.7	1.1691	Thr716Ile
HLA-DQA10201-DQB10202	716	**TNFTISVTTEILPVS**	0.5562	121.8	1.1691	Thr716Ile
HLA-DQA10201-DQB10301	716	**TNFTISVTTEILPVS**	0.7923	9.5	1.1691	Thr716Ile
DRB1_0701	717	**NFTISVTTEILPVSM**	0.7886	9.8	1.2136	-
DRB1_0901	717	**NFTISVTTEILPVSM**	0.6977	26.3	1.2136	-
HLA-DQA10103-DQB10603	717	**NFTISVTTEILPVSM**	0.4789	280.8	1.2136	-
HLA-DQA10201-DQB10202	717	**NFTISVTTEILPVSM**	0.5414	142.9	1.2136	-
HLA-DQA10201-DQB10301	717	**NFTISVTTEILPVSM**	0.7908	9.6	1.2136	-
HLA-DQA10201-DQB10303	717	**NFTISVTTEILPVSM**	0.6532	42.6	1.2136	-
DRB1_0701	718	**FTISVTTEILPVSMT**	0.7548	14.2	1.2603	-
HLA-DQA10201-DQB10202	718	**FTISVTTEILPVSMT**	0.5019	219.2	1.2603	-
HLA-DQA10201-DQB10303	718	**FTISVTTEILPVSMT**	0.6664	37	1.2603	-
HLA-DQA10103-DQB10603	718	**FTISVTTEILPVSMT**	0.4776	284.9	1.2603	-
HLA-DQA10201-DQB10303	719	**TISVTTEILPVSMTK**	0.6668	36.8	1.1821	-
HLA-DQA10201-DQB10303	720	**ISVTTEILPVSMTKT**	0.6708	35.2	1.3252	-
HLA-DQA10201-DQB10303	721	**SVTTEILPVSMTKTS**	0.6542	42.2	1.2417	-
HLA-DQA10201-DQB10303	722	**VTTEILPVSMTKTSV**	0.6488	44.7	1.1697	-
HLA-DQA10103-DQB10603	722	**VTTEILPVSMTKTSV**	0.4804	276.4	1.1697	-
HLA-DQA10102-DQB10501	722	**VTTEILPVSMTKTSV**	0.7807	10.7	1.1697	-
HLA-DQA10501-DQB10303	722	**VTTEILPVSMTKTSV**	0.5914	83.20	1.1697	-
HLA-DQA10102-DQB10501	723	**TTEILPVSMTKTSVD**	0.7819	10.6	1.1883	-
HLA-DQA10501-DQB10303	723	**TTEILPVSMTKTSVD**	0.5869	87.30	1.1883	-
HLA-DQA10103-DQB10603	723	**TTEILPVSMTKTSVD**	0.4784	282.4	1.1883	-
HLA-DQA10103-DQB10603	724	**TEILPVSMTKTSVDC**	0.4875	256	1.4758	-
HLA-DQA10103-DQB10603	725	**EILPVSMTKTSVDCT**	0.4764	288.5	1.6349	-
HLA-DQA10102-DQB10501	724	**TEILPVSMTKTSVDC**	0.784	10.3	1.4758	-
HLA-DQA10102-DQB10501	725	**EILPVSMTKTSVDCT**	0.7709	11.9	1.6349	-
HLA-DQA10102-DQB10501	726	**ILPVSMTKTSVDCT** M	0.7668	12.5	1.5023	-
DRB4_0101	893	**ALQIPFAMQMAYRFN**	0.7058	24.1	1.0112	-
HLA-DQA10501-DQB10402	893	**ALQIPFAMQMAYRFN**	0.7465	15.5	1.0112	-
HLA-DQA10501-DQB10402	896	**IPFAMQMAYRFN** GIG	0.7120	22.6	1.2828	-
DRB1_0405	1058	**GVVFLHVTYVPAQEK**	0.6920	28.0	1.1043	-
HLA-DPA10103-DPB10601	1059	**GVVFLHVTYVPAQEK**	0.8557	4.8	1.1043	-
HLA-DPA10103-DPB10601	1060	**VVFLHVTYVPAQEKN**	0.8510	5.0	1.1720	-
DRB1_0405	1060	**VVFLHVTYVPAQEKN**	0.6921	28.0	1.1720	-
DRB1_0405	1060	**VFLHVTYVPAQEKN**F	0.6738	34.1	1.0339	-
HLA-DPA10103-DPB10201	1206	**YEQYIKWPWYIWLGF**	0.7210	20.5	1.0674	-
HLA-DQA10601-DQB10402	1206	**YEQYIKWPWYIWLGF**	0.5292	163.0	1.0674	-
HLA-DPA10103-DPB10201	1207	**EQYIKWPWYIWLGFI**	0.7240	19.8	1.0096	-
HLA-DQA10601-DQB10402	1208	**QYIKWPWYIWLGFIA**	0.5235	173.5	1.1541	-
HLA-DPA10103-DPB10201	1208	**QYIKWPWYIWLGFIA**	0.7183	21.1	1.1541	-
DRB1_1201	1259	DDSEPVLKGVKLHYT	0.6238	58.6	1.1849	-

### Calculating the conservation of epitopes

The S protein showed a noticeably high percentage of conservancy based on the epitope conservancy study for the B-cell, CTL, and HTL selected epitopes. Specifically, conservancy rates for B-cell, CTL, and HTL epitopes ranged from 99% to 100%, 85% to 100%, and 81% to 100%, respectively. Tables 5–7 present comprehensive results regarding epitope conservation. Among the four proteins, the S protein showed the highest epitope conservation while the E protein had the lowest. These results suggest that the chosen epitopes are highly conserved.

**Table 5:** Conservation of B-cell epitopes.

Epitope sequence	Epitope length	Percent of protein sequence matches at identity <= 100%	Min. identity	Max. identity	Allergenicity	Toxicity	Antigenicity
SQCVNFRTRTQLPSAYTNSFTRGVY	25	0.00% (0/100)	84.00%	92.00%	Non-Allergenic	Non-Toxic	0.4268
FSNVTWFHAIHVSGTNGTKRFDN	23	97.00% (97/100)	95.65%	100.00%	Potentially Allergenic	Non-Toxic	0.6767
YPFLDVYHHKNNKSWME	17	0.00% (0/100)	76.47%	82.35%	Potentially Allergenic	Non-Toxic	0.4813
MDLEGKQGNFKNL	13	100.00% (100/100)	100.00%	100.00%	Non-Allergenic	Non-Toxic	1.2592
KHTPINLGRDLPQGFS	16	0.00% (0/100)	87.50%	93.75%	Non-Allergenic	Non-Toxic	1.0217
TPGDSSSGWTA	11	99.00% (99/100)	90.91%	100.00%	Non-Allergenic	Non-Toxic	0.2473
KSFTVEKGIYQTSNFRVQP	19	98.00% (98/100)	94.74%	100.00%	Non-Allergenic	Non-Toxic	0.5729
FPNITNLCPFDEVFNATKFASVYAWNRKRISNCVA	35	0.00% (0/100)	91.43%	94.29%	Potentially Allergenic	Non-Toxic	0.5066
YNFAHFFAFKCYGVSPTKLNDLCFT	25	0.00% (0/100)	84.00%	84.00%	Potentially Allergenic	Non-Toxic	1.5928
GNEVSQMAPGQTGNIADYNYKLP	23	0.00% (0/100)	82.61%	82.61%	Non-Allergenic	Non-Toxic	0.8444
KLDSKVGSNYNYRYRLFRKSNLKPFERDISTEIYQAGNKPCNGVAGVNCYFPLRSYSFRPTY	62	0.00% (0/100)	80.65%	83.87%	Potentially Allergenic	Non-Toxic	0.5843
ELLHAPATVCGPKKSTNLVKN	21	97.00% (97/100)	95.24%	100.00%	Non-Allergenic	Non-Toxic	0.0029
SNKKFLPF	8	99.00% (99/100)	87.50%	100.00%	Non-Allergenic	Non-Toxic	1.3952
NCTEVPVAIHADQLTPT	17	99.00% (99/100)	94.12%	100.00%	Non-Allergenic	Non-Toxic	0.3987
RVYSTGSNVFQ	11	99.00% (99/100)	90.91%	100.00%	Non-Allergenic	Non-Toxic	−0.1000
YVNNSYECDIPI	12	0.00% (0/100)	91.67%	91.67%	Non-Allergenic	Non-Toxic	0.5694
ASYQTQTNSRRRARSVASQ	19	0.00% (0/100)	89.47%	94.74%	Non-Allergenic	Non-Toxic	0.6720
YTMSLGVENSVAYSNN	16	0.00% (0/100)	93.75%	93.75%	Non-Allergenic	Non-Toxic	0.6467
EQDKNTK	7	0.00% (0/100)	85.71%	85.71%	Non-Allergenic	Non-Toxic	−0.2018
KQIYKTPPIKYFGGF	15	0.00% (0/100)	93.33%	93.33%	Potentially Allergenic	Non-Toxic	−0.6262
PDPSKPSK	8	99.00% (99/100)	87.50%	100.00%	Non-Allergenic	Non-Toxic	0.0621
LADAGFIKQYGDCLG	15	98.00% (98/100)	93.33%	100.00%	Non-Allergenic	Non-Toxic	0.2071
GQSKRVDFC	9	100.00% (100/100)	100.00%	100.00%	Non-Allergenic	Non-Toxic	1.7790
RNFYEPQIITTH	12	0.00% (0/100)	83.33%	91.67%	Non-Allergenic	Non-Toxic	0.3349
VNNTVYDPLQPELESFKEELDKYFKNHTSPDVDLGDISGI	40	0.00% (0/100)	95.00%	97.50%	Potentially Allergenic	Non-Toxic	0.2426
SCCKFDEDDSEPVLKG	16	99.00% (99/100)	93.75%	100.00%	Non-Allergenic	Non-Toxic	0.4347

**Table 6:** Conservation of CTL epitopes.

Epitope sequence	Epitope length	Percent of protein sequence matches at identity <= 100%	Minimum identity	Maximum identity	Allergenicity	Toxicity	Antigenicity
VNLRRRTQL	9	0.00% (0/100)	66.67%	88.89%	Non-Allergenic	Non-Toxic	1.7433
LRTRTQLPP	9	1.00% (1/100)	77.78%	100.00%	Non-Allergenic	Non-Toxic	1.4299
RTRTQLPPA	9	1.00% (1/100)	77.78%	100.00%	Non-Allergenic	Non-Toxic	1.2776
TRTQLPPAY	9	96.00% (96/100)	88.89%	100.00%	Non-Allergenic	Non-Toxic	1.2923
LPFFSNVTW	9	100.00% (100/100)	100.00%	100.00%	Potentially Allergenic	Non-Toxic	1.0808
HVSGTNGTK	9	97.00% (97/100)	88.89%	100.00%	Non-Allergenic	Non-Toxic	1.0956
FASIEKSNI	9	0.00% (0/100)	88.89%	88.89%	Non-Allergenic	Non-Toxic	1.0011
TLDSKTQSL	9	100.00% (100/100)	100.00%	100.00%	Non-Allergenic	Non-Toxic	1.0685
YSKHTPINL	9	100.00% (100/100)	100.00%	100.00%	Non-Allergenic	Non-Toxic	1.0547
SKHTPINLV	9	100.00% (100/100)	100.00%	100.00%	Non-Allergenic	Non-Toxic	1.1051
LPIGINITR	9	100.00% (100/100)	100.00%	100.00%	Non-Allergenic	Non-Toxic	1.8215
YYVGYLQPR	9	100.00% (100/100)	100.00%	100.00%	Potentially Allergenic	Non-Toxic	1.4692
PLSETKCTL	9	100.00% (100/100)	100.00%	100.00%	Non-Allergenic	Non-Toxic	1.0147
VRFPNITNL	9	100.00% (100/100)	100.00%	100.00%	Non-Allergenic	Non-Toxic	1.1141
RFPNITNLC	9	100.00% (100/100)	100.00%	100.00%	Non-Allergenic	Non-Toxic	1.2172
FPNITNLCP	9	100.00% (100/100)	100.00%	100.00%	Non-Allergenic	Non-Toxic	1.6218
KCYGVSPTK	9	100.00% (100/100)	100.00%	100.00%	Non-Allergenic	Non-Toxic	1.4199
CYGVSPTKL	9	100.00% (100/100)	100.00%	100.00%	Non-Allergenic	Non-Toxic	1.4263
LNDLCFTNV	9	99.00% (99/100)	88.89%	100.00%	Non-Allergenic	Non-Toxic	2.0179
EVRQIAPGQ	9	100.00% (100/100)	100.00%	100.00%	Non-Allergenic	Non-Toxic	1.1205
RQIAPGQTG	9	100.00% (100/100)	100.00%	100.00%	Non-Allergenic	Non-Toxic	1.7890
KIADYNYKL	9	100.00% (100/100)	100.00%	100.00%	Non-Allergenic	Non-Toxic	1.6639
IADYNYKLP	9	100.00% (100/100)	100.00%	100.00%	Non-Allergenic	Non-Toxic	1.1012
KVGGNYNYL	9	100.00% (100/100)	100.00%	100.00%	Non-Allergenic	Non-Toxic	0.5994
VGGNYNYLY	9	99.00% (99/100)	88.89%	100.00%	Potentially Allergenic	Non-Toxic	0.7432
GGNYNYLYR	9	99.00% (99/100)	88.89%	100.00%	Potentially Allergenic	Non-Toxic	0.0647
GNYNYLYRL	9	99.00% (99/100)	88.89%	100.00%	Potentially Allergenic	Non-Toxic	0.1170
GFACYFPLQ	9	0.00% (0/100)	77.78%	88.89%	Potentially Allergenic	Non-Toxic	1.1408
YGFRPTNGV	9	0.00% (0/100)	88.89%	88.89%	Non-Allergenic	Non-Toxic	1.1496
VGHQPYRVV	9	0.00% (0/100)	77.78%	88.89%	Non-Allergenic	Non-Toxic	0.9709
PYRVVVLSF	9	99.00% (99/100)	88.89%	100.00%	Potentially Allergenic	Non-Toxic	1.0281
RVVVLSFEL	9	100.00% (100/100)	100.00%	100.00%	Non-Allergenic	Non-Toxic	1.1918
VVLSFELLH	9	100.00% (100/100)	100.00%	100.00%	Non-Allergenic	Non-Toxic	1.4090
LVKNKCVNF	9	100.00% (100/100)	100.00%	100.00%	Non-Allergenic	Non-Toxic	1.3263
KNKCVNFNF	9	100.00% (100/100)	100.00%	100.00%	Non-Allergenic	Non-Toxic	2.4991
CVNFNFNGL	9	100.00% (100/100)	100.00%	100.00%	Non-Allergenic	Non-Toxic	1.7985
ESNKKFLPF	9	99.00% (99/100)	88.89%	100.00%	Non-Allergenic	Non-Toxic	1.0278
DIDDTDDAV	9	0.00% (0/100)	77.78%	77.78%	Non-Allergenic	Non-Toxic	1.1224
ILDITPCSF	9	100.00% (100/100)	100.00%	100.00%	Non-Allergenic	Non-Toxic	1.1835
ITPCSFGGV	9	100.00% (100/100)	100.00%	100.00%	Non-Allergenic	Non-Toxic	1.3871
CSFGGVSVI	9	99.00% (99/100)	88.89%	100.00%	Non-Allergenic	Non-Toxic	1.2630
YQGVNCTEV	9	0.00% (0/100)	88.89%	88.89%	Non-Allergenic	Non-Toxic	1.3957
GVNCTEVPV	9	0.00% (0/100)	88.89%	88.89%	Non-Allergenic	Non-Toxic	1.0848
QLTPTWRVY	9	100.00% (100/100)	100.00%	100.00%	Non-Allergenic	Non-Toxic	1.2119
LTPTWRVYS	9	100.00% (100/100)	100.00%	100.00%	Non-Allergenic	Non-Toxic	1.1258
VFQTRAGCL	9	100.00% (100/100)	100.00%	100.00%	Non-Allergenic	Non-Toxic	1.7094
FQTRAGCLI	9	100.00% (100/100)	100.00%	100.00%	Non-Allergenic	Non-Toxic	1.7332
NSPRRARSV	9	99.00% (99/100)	88.89%	100.00%	Non-Allergenic	Non-Toxic	0.1034
SRRRARSVA	9	0.00% (0/100)	77.78%	88.89%	Non-Allergenic	Non-Toxic	1.4417
PRRARSVAS	9	98.00% (98/100)	88.89%	100.00%	Non-Allergenic	Non-Toxic	0.4829
EILPVSMTK	9	99.00% (99/100)	88.89%	100.00%	Non-Allergenic	Non-Toxic	1.6842
LPVSMTKTS	9	99.00% (99/100)	88.89%	100.00%	Non-Allergenic	Non-Toxic	1.5550
KTSVDCTMY	9	100.00% (100/100)	100.00%	100.00%	Non-Allergenic	Non-Toxic	1.1824
LLLQYGSFC	9	100.00% (100/100)	100.00%	100.00%	Potentially Allergenic	Non-Toxic	1.3260
QYGSFCTQL	9	100.00% (100/100)	100.00%	100.00%	Non-Allergenic	Non-Toxic	1.2906
LQIPFAMQM	9	100.00% (100/100)	100.00%	100.00%	Non-Allergenic	Non-Toxic	1.0680
IPFAMQMAY	9	100.00% (100/100)	100.00%	100.00%	Non-Allergenic	Non-Toxic	1.4278
PFAMQMAYR	9	100.00% (100/100)	100.00%	100.00%	Non-Allergenic	Non-Toxic	1.3315
FAMQMAYRF	9	100.00% (100/100)	100.00%	100.00%	Non-Allergenic	Non-Toxic	1.0278
AYRFNGIGV	9	100.00% (100/100)	100.00%	100.00%	Non-Allergenic	Non-Toxic	1.2995
YRFNGIGVT	9	100.00% (100/100)	100.00%	100.00%	Non-Allergenic	Non-Toxic	1.7692
GVVFLHVTY	9	100.00% (100/100)	100.00%	100.00%	Non-Allergenic	Non-Toxic	1.4104
VVFLHVTYV	9	100.00% (100/100)	100.00%	100.00%	Non-Allergenic	Non-Toxic	1.5122
FLHVTYVPA	9	99.00% (99/100)	88.89%	100.00%	Non-Allergenic	Non-Toxic	1.3346
EQYIKWPWY	9	100.00% (100/100)	100.00%	100.00%	Potentially Allergenic	Non-Toxic	1.1384
QYIKWPWYI	9	100.00% (100/100)	100.00%	100.00%	Potentially Allergenic	Non-Toxic	1.4177
KWPWYIWLG	9	100.00% (100/100)	100.00%	100.00%	Potentially Allergenic	Non-Toxic	1.0478
WPWYIWLGF	9	100.00% (100/100)	100.00%	100.00%	Potentially Allergenic	Non-Toxic	1.4953
WYIWLGFIA	9	100.00% (100/100)	100.00%	100.00%	Potentially Allergenic	Non-Toxic	1.0356
IAIVMVTIM	9	98.00% (98/100)	88.89%	100.00%	Potentially Allergenic	Non-Toxic	1.1339
EPVLKGVKL	9	100.00% (100/100)	100.00%	100.00%	Non-Allergenic	Non-Toxic	1.2301
VLKGVKLHY	9	100.00% (100/100)	100.00%	100.00%	Non-Allergenic	Non-Toxic	1.2378

**Table 7:** Conservation of HTL epitopes.

Epitope sequence	Epitope length	Percent of protein sequence matches at identity <= 100%	Min. identity	Max. identity	Allergenicity	Toxicity	Antigenicity
FVLLPLVSSQCVNFR	15	0.00% (0/100)	73.33%	86.67%	Potentially Allergenic	Non-Toxic	1.1998
LLPLVSSQCVNFRTR	15	0.00% (0/100)	80.00%	93.33%	Non-Allergenic	Non-Toxic	1.3347
TAGAAAYYVGYLQPR	15	98.00% (98/100)	93.33%	100.00%	Potentially Allergenic	Non-Toxic	1.0413
FFTFKCYGVSPTKLN	15	0.00% (0/100)	93.33%	93.33%	Non-Allergenic	Non-Toxic	1.0413
FAFKCYGVSPTKLND	15	0.00% (0/100)	86.67%	86.67%	Non-Allergenic	Non-Toxic	1.7009
KLNDLCFTNVYADSF	15	99.00% (99/100)	93.33%	100.00%	Non-Allergenic	Non-Toxic	1.1791
NCYFPLQSYGFQPTN	15	100.00% (100/100)	100.00%	100.00%	Potentially Allergenic	Non-Toxic	1.0385
GVGHQPYRVVVLSFE	15	0.00% (0/100)	86.67%	93.33%	Non-Allergenic	Non-Toxic	1.1163
VGHQPYRVVVLSFEL	15	0.00% (0/100)	86.67%	93.33%	Non-Allergenic	Non-Toxic	1.1362
GHQPYRVVVLSFELL	15	0.00% (0/100)	86.67%	93.33%	Non-Allergenic	Non-Toxic	0.8842
KCVNFNFNGLKGTGV	15	0.00% (0/100)	93.33%	93.33%	Non-Allergenic	Non-Toxic	1.4448
CVNFNFNGLKGTGVL	15	0.00% (0/100)	93.33%	93.33%	Non-Allergenic	Non-Toxic	1.2222
VNFNFNGLKGTGVLT	15	0.00% (0/100)	86.67%	93.33%	Non-Allergenic	Non-Toxic	1.1380
NFNFNGLKGTGVLTE	15	0.00% (0/100)	86.67%	93.33%	Non-Allergenic	Non-Toxic	0.9457
LQYGSFCTQLNRALT	15	100.00% (100/100)	100.00%	100.00%	Non-Allergenic	Non-Toxic	1.0270
PQTLEILDITPCSFG	15	100.00% (100/100)	100.00%	100.00%	Non-Allergenic	Non-Toxic	1.5270
QTLEILDITPCSFGG	15	100.00% (100/100)	100.00%	100.00%	Non-Allergenic	Non-Toxic	1.4946
TLEILDITPCSFGGV	15	100.00% (100/100)	100.00%	100.00%	Non-Allergenic	Non-Toxic	1.6214
DITPCSFGGVSVITP	15	99.00% (99/100)	93.33%	100.00%	Non-Allergenic	Non-Toxic	1.2254
PINFTISVTTEILPV	15	0.00% (0/100)	86.67%	93.33%	Non-Allergenic	Non-Toxic	1.4167
INFTISVTTEILPVS	15	0.00% (0/100)	86.67%	93.33%	Non-Allergenic	Non-Toxic	1.3924
NFTISVTTEILPVSM	15	99.00% (99/100)	93.33%	100.00%	Non-Allergenic	Non-Toxic	1.2136
FTISVTTEILPVSMT	15	99.00% (99/100)	93.33%	100.00%	Non-Allergenic	Non-Toxic	1.2603
TISVTTEILPVSMTK	15	99.00% (99/100)	93.33%	100.00%	Non-Allergenic	Non-Toxic	1.1821
ISVTTEILPVSMTKT	15	99.00% (99/100)	93.33%	100.00%	Non-Allergenic	Non-Toxic	1.3252
SVTTEILPVSMTKTS	15	99.00% (99/100)	93.33%	100.00%	Non-Allergenic	Non-Toxic	1.2417
VTTEILPVSMTKTSV	15	99.00% (99/100)	93.33%	100.00%	Non-Allergenic	Non-Toxic	1.1697
TTEILPVSMTKTSVD	15	99.00% (99/100)	93.33%	100.00%	Non-Allergenic	Non-Toxic	1.1883
EILPVSMTKTSVDCT	15	99.00% (99/100)	93.33%	100.00%	Non-Allergenic	Non-Toxic	1.6349
TEILPVSMTKTSVDC	15	99.00% (99/100)	93.33%	100.00%	Non-Allergenic	Non-Toxic	1.4758
ILPVSMTKTSVDCTM	15	99.00% (99/100)	93.33%	100.00%	Non-Allergenic	Non-Toxic	1.5023
ALQIPFAMQMAYRFN	15	99.00% (99/100)	93.33%	100.00%	Potentially Allergenic	Non-Toxic	1.0112
IPFAMQMAYRFNGIG	15	100.00% (100/100)	100.00%	100.00%	Potentially Allergenic	Non-Toxic	1.2828
GVVFLHVTYVPAQEK	15	99.00% (99/100)	93.33%	100.00%	Non-Allergenic	Non-Toxic	1.1043
VVFLHVTYVPAQEKN	15	99.00% (99/100)	93.33%	100.00%	Non-Allergenic	Non-Toxic	1.1720
VFLHVTYVPAQEKNF	15	99.00% (99/100)	93.33%	100.00%	Non-Allergenic	Non-Toxic	1.0339
YEQYIKWPWYIWLGF	15	100.00% (100/100)	100.00%	100.00%	Potentially Allergenic	Non-Toxic	1.0674
EQYIKWPWYIWLGFI	15	100.00% (100/100)	100.00%	100.00%	Potentially Allergenic	Non-Toxic	1.0096
QYIKWPWYIWLGFIA	15	100.00% (100/100)	100.00%	100.00%	Potentially Allergenic	Non-Toxic	1.1541
DDSEPVLKGVKLHYT	15	99.00% (99/100)	93.33%	100.00%	Non-Allergenic	Non-Toxic	1.1849

### Population coverage

**Fig 3:**
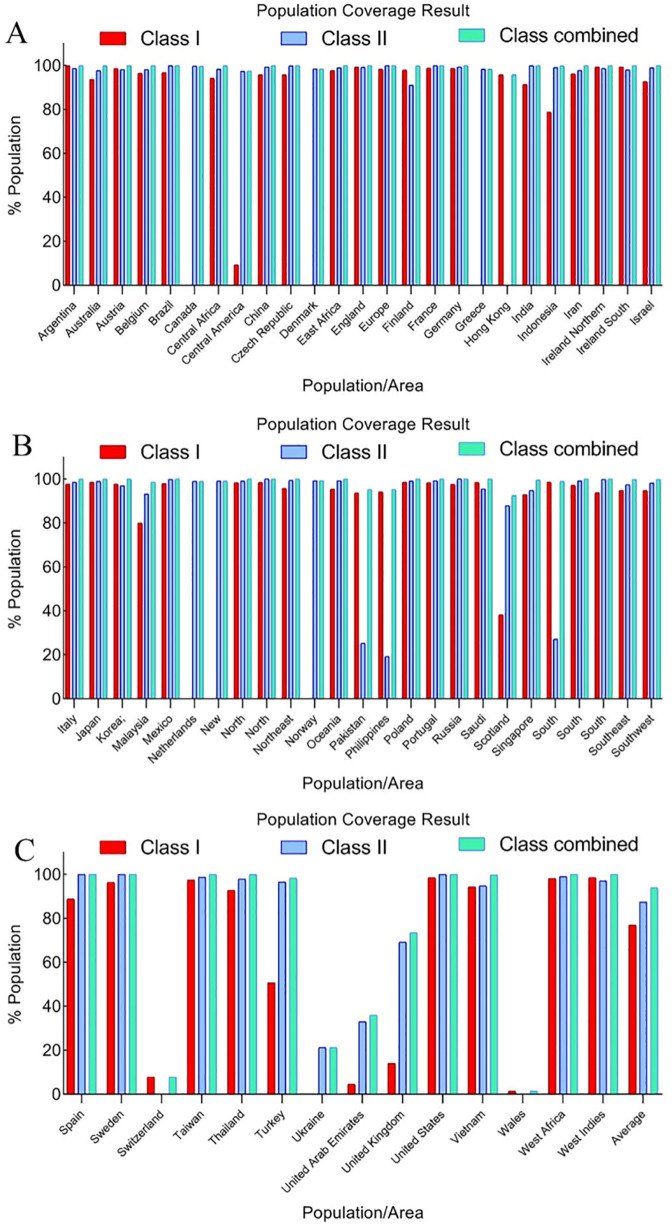
Comprehensive population coverage analysis of selected epitopes across different geographical regions. The bar charts illustrate the predicted population coverage of selected MHC Class I (red), Class II (blue), and combined (cyan) epitopes across various countries and regions based on HLA allele frequencies. This analysis was performed using the IEDB population coverage tool to evaluate the potential global applicability and effectiveness of the designed multi-epitope vaccine constructs (Cov19-B and Cov19-T). The high levels of combined population coverage observed in most regions (>80%) indicate broad immunogenic potential and support the suitability of the vaccine candidates in diverse genetic backgrounds.

For every selected epitope, population coverage was evaluated based on its frequency among different ethnic groups. MHC I, MHC II, and the combined MHC I–MHC II class alleles were considered in the analysis. With an average of 93.8%, the combined class alleles showed the highest population coverage for epitopes overall (S1 Table). As shown in Fig 3A, B and C, it can be seen that the chosen epitopes were present in the populations in 64 different regions.

### Prediction of B-cell epitopes: Conformational

Additionally, conformational B-cell epitopes from the final structures of Cov19B and Cov19T were analyzed, focusing on identifying any overlap between different types of B-cell epitopes (S2 and S3 Tables)

### Construction analyzation

**Fig 4:**
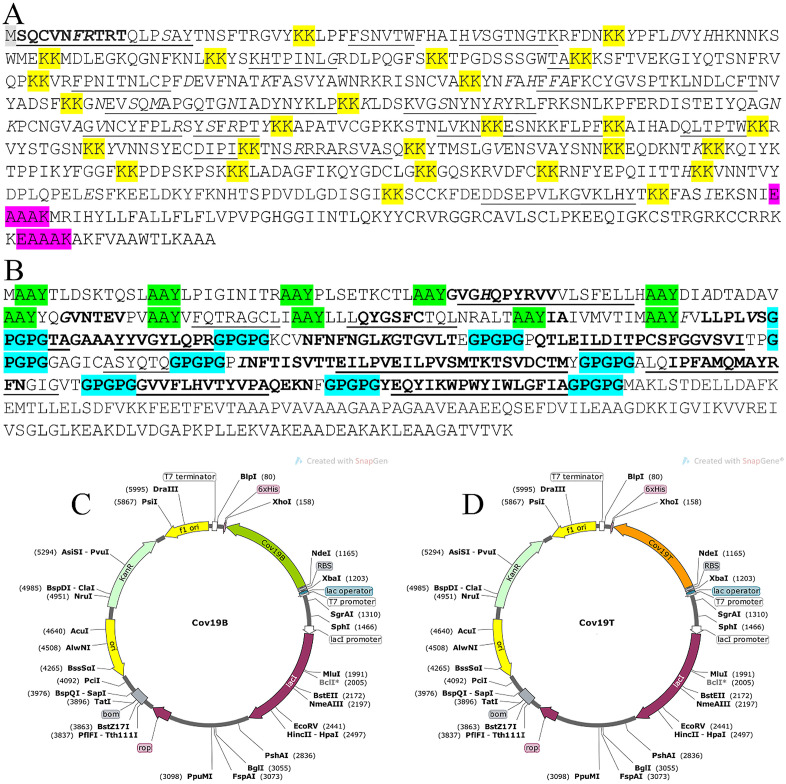
Schematic representation of vaccine constructs Cov19B and Cov19T, including linker arrangements, adjuvants, and in silico cloning. (A) Amino acid sequence of the Cov19B vaccine construct showing the arrangement of selected B-cell, CTL, and HTL epitopes linked with appropriate peptide linkers (highlighted in yellow: KK, pink: EAAAK, gray: epitope regions). The sequence includes the PADRE sequence and the Human Beta-defensin 3 (HBD3) as adjuvants. (B) Cov19T construct sequence featuring similar epitope-linker architecture. The CTL and HTL epitopes are joined by GPGPG linkers (cyan), and the AAY linkers (green) connect cytotoxic epitopes to preserve MHC-I processing efficiency. The PADRE and L7/L12 ribosomal protein from M. tuberculosis serve as adjuvants. (C) In silico cloning of Cov19B into the pET26b(+) expression vector using restriction sites NdeI and XhoI, confirming suitability for prokaryotic expression systems. (D) Simulated cloning of Cov19T into the same vector system. Both constructs were codon-optimized and checked for restriction enzyme compatibility using SnapGene software to ensure efficient bacterial express.

For the first construct (Cov19B), 24 B-cell epitopes were fused using KK linkers. Human Beta-defensin 3 (HBD3) and PADRE sequences were linked with an EAAAK linker. For the second construct (Cov19T), 10 CTL and 8 HTL epitopes were linked via AAY and GPGPG linkers, respectively. The 50S ribosomal protein L7/L12 from *Mycobacterium tuberculosis* was fused as an adjuvant to the C-terminal of Cov19T via EAAAK linker (Fig 4A & 4B).

*XhoI* and *NdeI* restriction sites were incorporated into both Cov19-B and Cov19-T constructs. The vaccine’s 1899 base pair nucleotide sequence was optimized. Following codon optimization, analysis revealed GC-Content of 47.35% for Cov19-B and 57.30% for Cov19-T. These results indicate effective codon adaptation for efficient expression in the selected bacterial strain.

As shown, the constructs were cloned intopET26b(+) for *in-silico* cloning (Fig 4C & 4D). The restriction enzymes *Xho*I and *Nde*I were positioned at the construct’s N- and C-terminals, respectively. Similarly, the same restriction sites were placed at corresponding positions in Cov19-T. Furthermore, a HisTag sequence was added to the C-terminus of each construct. The constructs are in the frame, as demonstrated by examining the final nucleotide sequence performed.

The results of physico-chemical properties indicate that the constructs comprise two proteins with a 649 (Cov19-B) and 465 (Cov19-T) aa with 74 kDa and 48 kDa molecular weight. The theoretical isoelectric point (pI) was determined to be 9.95 and 8.49 for Cov19-B and Cov19-T, respectively. The instability index was calculated as 38.11 for Cov19-B and 23.09 for Cov19-T while the aliphatic index was estimated to be approximately 58.15 and 94.06, respectively, for Cov19-B and Cov19-T. Furthermore, the protein obtained a GRAVY of −0.785 (Cov19-B) and −0.295 (Cov19-T). These calculated physicochemical parameters collectively indicate that both Cov19-B and Cov19-T represent suitable constructs for expression.

### Homology modeling and its analyzation

**Fig 5:**
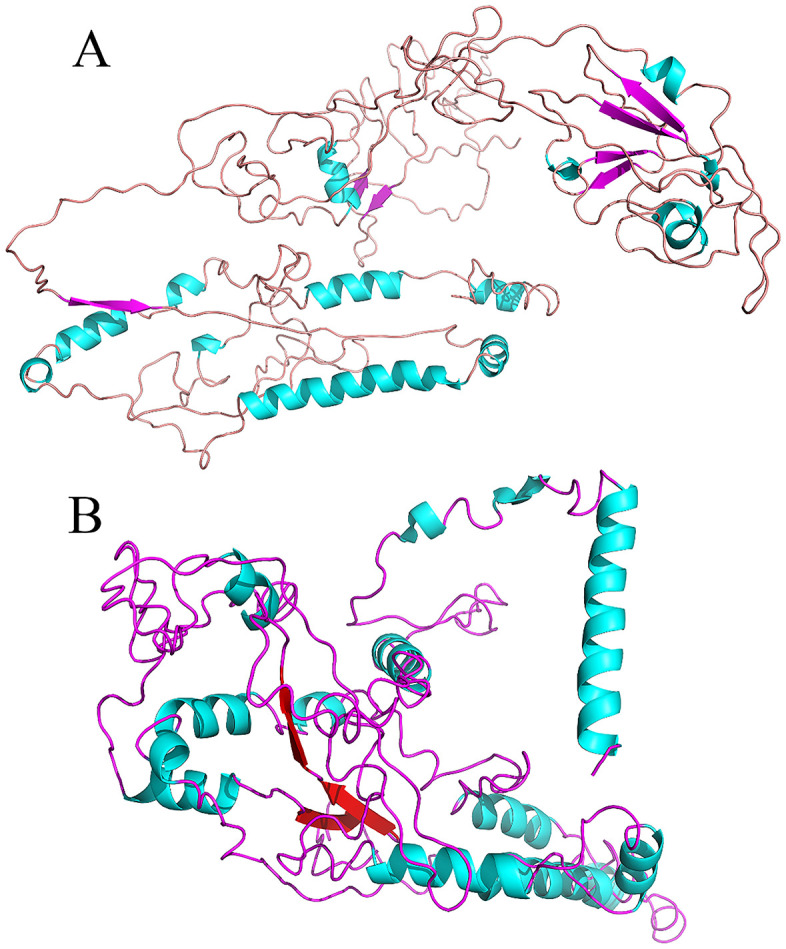
(A) Modeled 3D structure of Cov19B and (B) Modeled 3D structure of Cov19T.

The iterative refinement process enhances the precision and dependability of the predicted structure, leading to a clearer comprehension of the vaccine’s spatial arrangement and possible interactions with target molecules. As shown in Figs 5A and 5B, the amino acid sequence was estimated as the vaccine’s three-dimensional structure. Subsequently, the first 3D structure was refined, resulting in a final structure with a reduced root mean square deviation and improved structural features.

**Fig 6:**
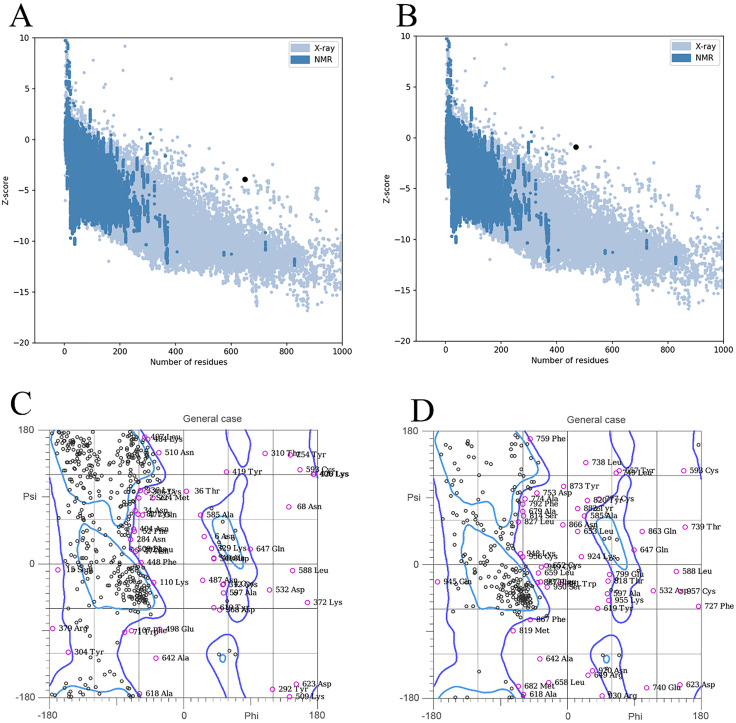
Validation of homology modeling for the multi-epitope vaccine constructs Cov19-B and Cov19-T, designed to target SARS-CoV-2. (A) Z-Score analysis of Cov19-B, calculated using ProSA-web, to assess the overall quality of the modeled structure. The Z-Score of −3.9, falls within the range of native protein structures, indicating high structural reliability and consistency with experimentally determined structures of similar size. (B) Z-Score analysis of Cov19-T, similarly computed, with a Z-Score of −0.9, confirming the model’s quality and structural integrity. (C) Ramachandran plot of Cov19-B, illustrating the distribution of phi (φ) and psi (ψ) angles of amino acid residues. The plot reveals 85% of residues in favored regions, indicating a high-quality model with minimal steric clashes. (D) Ramachandran plot of Cov19-T, showing 72% of residues in favored regions, further validating the structural accuracy of the modeled construct.

Z-score of −3.9 (Cov19B) and −0.9 (Cov19T) were found using additional validation criteria for the improved 3D structure of (Fig 6A and 6B). Figs 6C and 6D show the Ramachandran graph, which shows that 85% and 72% of amino acids are situated in the preferred and allowed regions, respectively.

Although the Ramachandran analysis showed that less than 90% of residues fell within the favored regions for the vaccine constructs, the values remained close to this benchmark and are within acceptable limits for multi-epitope synthetic designs containing numerous flexible linker regions and non-native adjuvant sequences. The structural models were further validated through Z-score evaluation (via SAVES), low MolProbity clashscores, and successful structural refinement using ModRefiner. Moreover, molecular docking and 100 ns molecular dynamics simulations confirmed the constructs’ stability and correct folding, supporting their suitability for experimental expression and immunogenicity studies

### Disulfide engineering of vaccine constructs

Disulfide engineering was performed to enhance the structural stability of Cov19-B and Cov19-T. The 3D structures were analyzed to identify residue pairs for disulfide bond formation. Four disulfide bonds were introduced into Cov19-B and two into Cov19-T by mutating spatially proximal residue pairs to cysteines. Pairs were selected based on a disulfide bond formation likelihood (%SS) ≥90%, bond energy ≤5 kcal/mol, and Σ β-Factor ≤15, ensuring stability. For Cov19-B, pairs A194 CYS–A199 PRO (χ3 = +99.50°, bond energy = 1.79 kcal/mol, Σ β-Factor = 8.34), A217 LYS–A221 VAL (χ3 = −76.06°, bond energy = 1.71 kcal/mol, Σ β-Factor = 8.47), A102 ASP–A105 GLN (χ3 = +74.45°, bond energy = 4.26 kcal/mol, Σ β-Factor = 10.16) and A166 ALA–A172 ASN (χ3 = −92.79°, bond energy = 0.54 kcal/mol, Σ β-Factor = 8.57) were selected (S1 Fig).

The pairs A81 ALA–A90 ARG (χ3 = +102.00°, bond energy = 0.20 kcal/mol, Σ β-Factor = 15.42), and A134 ALA–A146 TYR (χ3 = +93.26°, bond energy = 1.40 kcal/mol, Σ β-Factor = 21.20) were selected for Cov19-T (S2 Fig). Post-engineering, both constructs retained high antigenicity, were non-allergenic, and non-toxic. Molecular docking confirmed that disulfide bonds did not disrupt Cov19-B or Cov19-T binding affinities (ΔG < −10 kcal/mol).

### Protein-protein docking

The structures of Cov19B and Cov19T were docked with HNA using the HDOCK server to identify interaction regions through protein-protein docking. The highest rankings complexes were chosen. The docking results revealed that Cov19B and Cov19T can interact with human-neutralizing antibodies (HNA). All Four complexes have negative binding energy (S4, S5, S6, and S7 Tables). So, in Cov19B-Heavy chain, Cov19B-Light, Cov19T-Heavy chain, and Cov19T-Light chain complexes had docking scores of −332.07, −323.14, −328.47, and −310.24, respectively.

**Fig 7:**
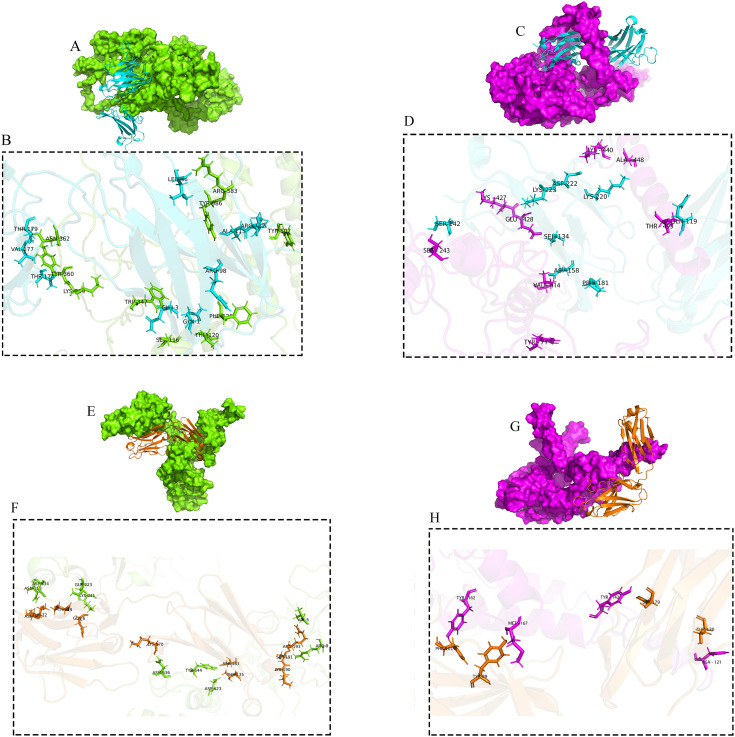
Docking visualization of Cov19B-Heavy chain, Cov19B-Light chain, Cov19T-Heavy chain and Cov19T-Light chain. (A) Cov19B structure in green color surface mode and Heavy chain in blue color cartoon mode. (B) Magnified interactions of Cov19B-Heavy chain. (C) Cov19B structure in green color surface mode and Light chain in copper color cartoon mode. (D) Magnified interactions of Cov19B-Light chain. (E) Cov19B structure in purple color surface mode and Heavy chain in blue color cartoon mode. (F) Magnified interactions of Cov19T-Heavy chain. (G) Cov19T structure in purple color surface mode and Light chain in copper color cartoon mode. (H) Magnified interactions of Cov19T-Light chain.

As demonstrated in Figs 7A and 7C, both Cov19B exhibits remarkable capacity to interact with the heavy and light chains of the HNA antibody. Notably, interactions take place between the HNA antibody’s heavy chain’s HCDR1, HCDR2, and HCDR3 loops as well as its light chain’s LCDR1, LCDR2, and LCDR3 loops. These regions were specifically selected due to their potential to bind diverse sections of the RBD antigen in both Cov19B and Cov19T. These interactions are essential for coordinating the intricate molecular interactions required for the identification of antibodies by antigens, which in turn prepares the immune system to mount a successful defense against the pathogen of interest. As shown in Figs 7B and 7D, the interaction involved amino Tyr856, Tyr507, Lys359, Tyr360, Asn362, Arg583, Phe126, Thr120, Ser116 and Trp 347 from Cov19B with amino acids Ala113, Arg112, Thr147, Val177, Thr179, Leu46, Arg98, Gln1 and Gln3 from the heavy chain of the HNA antibody. Additionally, amino acids Asn 263, Tyr644, Asp623, Asp234, Gln223, Lys241, Val5, Arg8 and Asn636 from Cov19B interacted with amino acids Asn71, Ser183, Thr135, Thr72, Thr19, Arg193, Gln6, Ser191, Lys190, Lys170 from the light chain of the HNA antibody.

For the Cov19T-Light chain complex, structural analysis revealed 7 hydrogen bonds and 1 salt bridge mediating interactions between Ala448, Lys440, Lys427, Tyr71, Glu428, Val414, Thr495, and Ser243 amino acids of Cov19T and Lys220, Asp222, Lys223, Ser186, Ser134, Asp158, Gln119, and Ser142 amino acids of Light chain (Fig 7E and 7G). The interaction in the Cov19T-Light chain complex exhibited 4 hydrogen bonds responsible for the interaction between Tyr162, Tyr142, Asn121 and Met167 of Cov19T and Phe101, Ser179, Thr120 and Tyr89 of light chain (Fig 7F and 7H).

**Fig 8:**
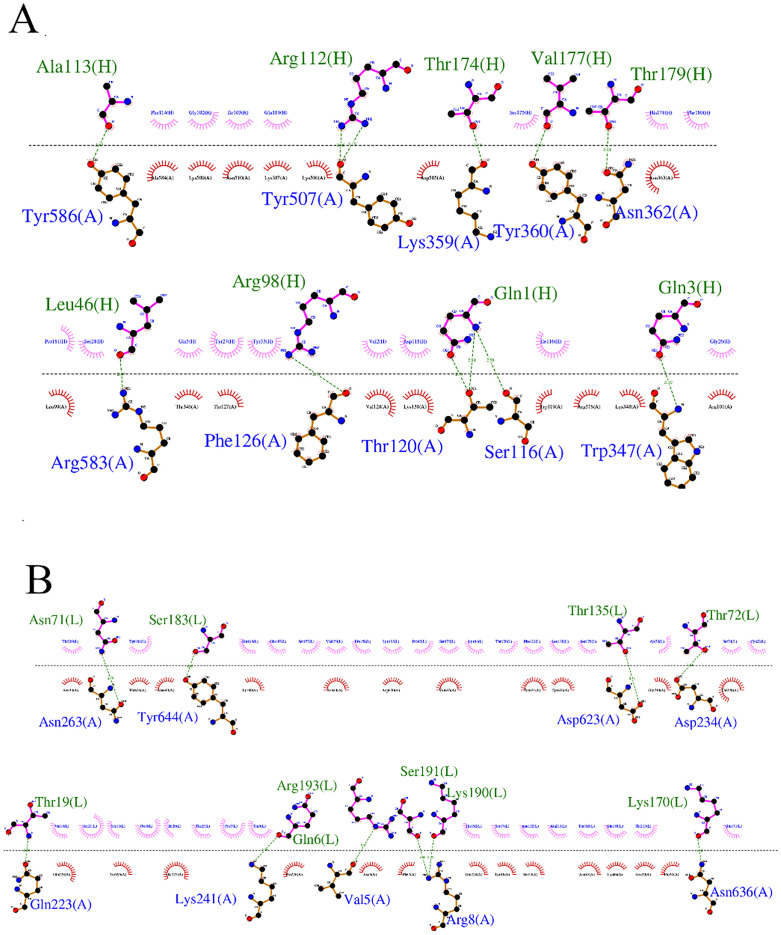
(A) 2D presentation of interactions in Cov19B-Heavy chain complex. (B) 2D presentation of interactions in Cov19B-Light chain complex.

**Fig 9:**
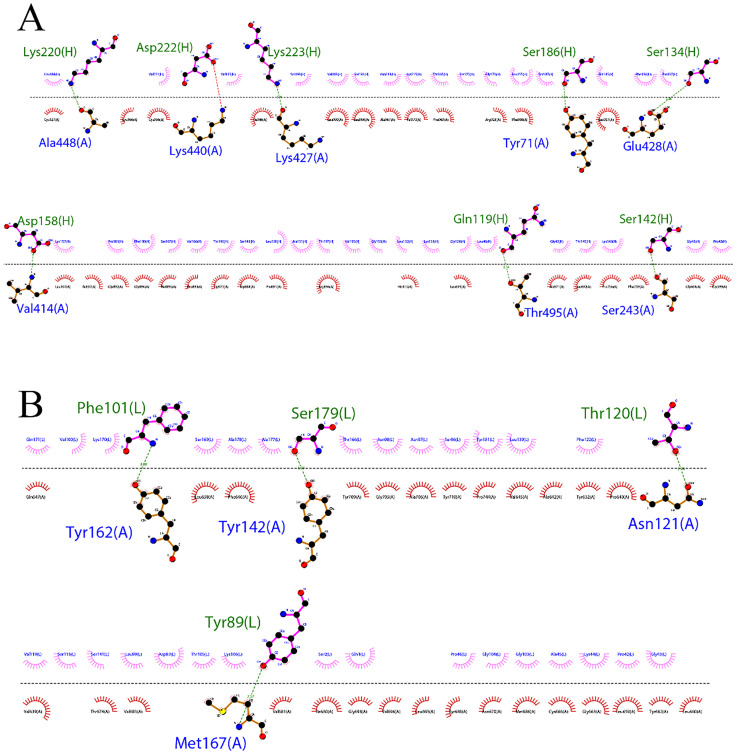
(A) 2D presentation of interactions in Cov19T-Heavy chain complex. (B) 2D presentation of interactions in Cov19T-Light chain complex.

The 2D interactions are summarized and visualized so that graphical 2D HDOCK results are presented in Fig 8A (Cov19B-Heavy chain) and 8B (Cov19B-Light chain), as well as Fig 9A (Cov19T-Heavy chain) and 9B (Cov19T-Light chain) offering a detailed view of the protein-protein docking outcomes.

### Analysis molecular dynamic and Poisson–Boltzmann Born surface area

**Fig 10:**
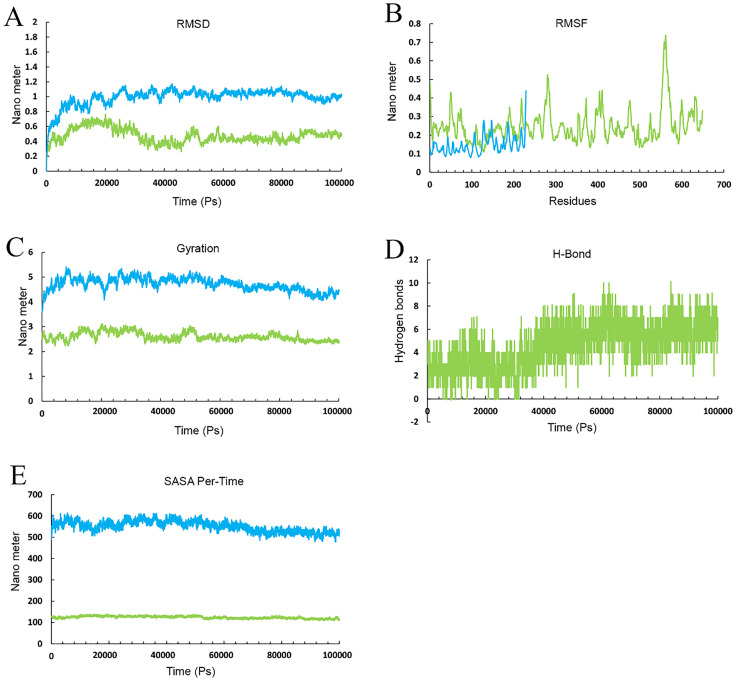
Molecular dynamics (MD) simulation results for the Cov19B-Heavy chain complex, evaluated over a 100 ns trajectory using GROMACS to assess structural stability and interaction dynamics. (A) Root Mean Square Deviation (RMSD) plot, showing the backbone deviation of Cov19B (green) and Heavy chain (blue) over time. Cov19B stabilizes at 0.4 nm after 40 ns, while the Heavy chain reaches equilibrium at 1 nm after 20 ns, indicating stable complex formation. (B) Root Mean Square Fluctuation (RMSF) plot, depicting residue-level flexibility. Cov19B exhibits low fluctuations 0.3 in epitope regions, while the Heavy chain shows higher fluctuations 0.22 nm in non-binding regions, suggesting conformational flexibility. (C) Radius of Gyration (Rg) plot, measuring the compactness of the complex. Both Cov19B and Heavy chain maintain stable Rg values 5 nm for Cov19B and 2.8 for Heavy chain, confirming structural integrity throughout the simulation. (D) Hydrogen bond (H-Bond) analysis, illustrating the number of H-bonds between Cov19B and Heavy chain over time. An average of 2–8 H-bonds is maintained, highlighting strong intermolecular interactions. (E) Solvent Accessible Surface Area (SASA) per-time plot, showing the solvent exposure of Cov19B (green) and Heavy chain (blue). SASA values stabilize at 500 nm for Cov19B and 100 nm² for Heavy chain, indicating minimal unfolding and consistent surface accessibility for immune recognition. These results collectively demonstrate the stability and interaction dynamics of the Cov19B-Heavy chain complex, supporting its potential for effective immune response elicitation.

**Fig 11:**
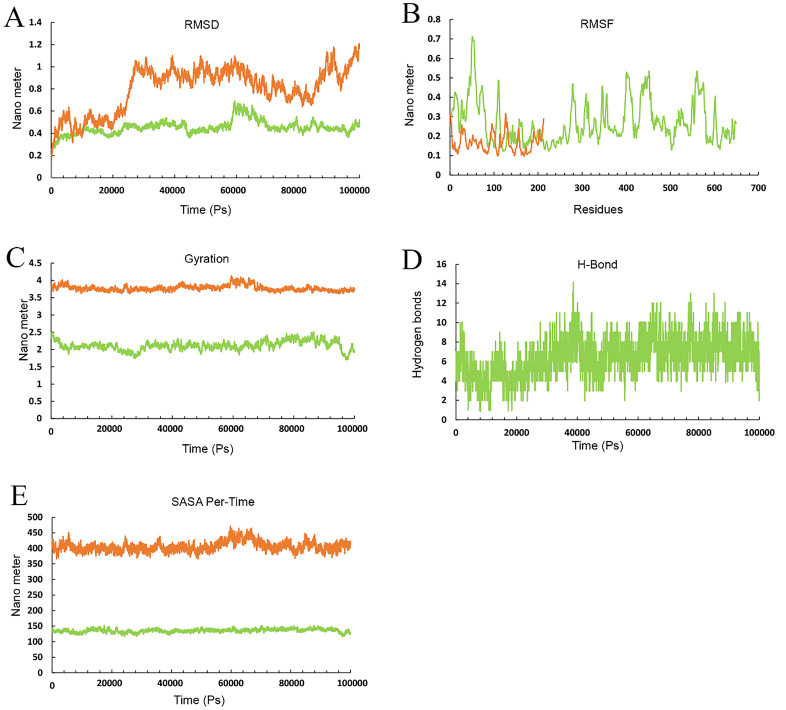
Molecular dynamics (MD) simulation results for the Cov19B-Light chain complex, evaluated over a 100 ns trajectory using GROMACS to assess structural stability and interaction dynamics. (A) Root Mean Square Deviation (RMSD) plot, showing the backbone deviation of Cov19B (green) and Light chain (Copper) over time. Cov19B stabilizes at 0.4 nm after 20 ns, while the Light chain reaches equilibrium at 1 nm after 30 ns, indicating stable complex formation. (B) Root Mean Square Fluctuation (RMSF) plot, depicting residue-level flexibility. Cov19B exhibits low fluctuations 0.25 in epitope regions, while the Light chain shows higher fluctuations 0.15 nm in non-binding regions, suggesting conformational flexibility. (C) Radius of Gyration (Rg) plot, measuring the compactness of the complex. Both Cov19B and Light chain maintain stable Rg values 3.8 nm for Cov19B and 2.3 for Light chain, confirming structural integrity throughout the simulation. (D) Hydrogen bond (H-Bond) analysis, illustrating the number of H-bonds between Cov19B and Light chain over time. An average of 4–10 H-bonds is maintained, highlighting strong intermolecular interactions. (E) Solvent Accessible Surface Area (SASA) per-time plot, showing the solvent exposure of Cov19B (green) and Light chain (Copper). SASA values stabilize at 400 nm² for Cov19B and 140 nm² for Light chain, indicating minimal unfolding and consistent surface accessibility for immune recognition. These results collectively demonstrate the stability and interaction dynamics of the Cov19B- Light chain complex, supporting its potential for effective immune response elicitation.

The docked results underwent MD simulation for 100 nanoseconds. The hydrogen bond in the Cov19B-Heavy chain complex and Cov19B-Light chain complex was formed with a Root Mean Square Deviation (RMSD) of 0.9 and 1 nm, respectively (Fig 10A and 11A). RMSF for the Cov19B-Heavy chain complex and Cov19B-Light chain complex indicated a bit volatility (Fig 10B and 11B). The radius of gyration related to the complexes in both graphs indicated that Cov19B was more stable than the Heavy chain and Light chain (Fig 10C and 11C). As we can see in Fig 10D and Fig 11D number of hydrogen bonds in the Cov19B-Light chain complex was more than those in the Cov19B-Heavy chain complex. SASA per-time graph also demonstrated that Cov19B showed a stable structure compared to Cov19B-Heavy chain complex and Cov19B-Light chain complex (Fig 10E and 11E).

**Fig 12:**
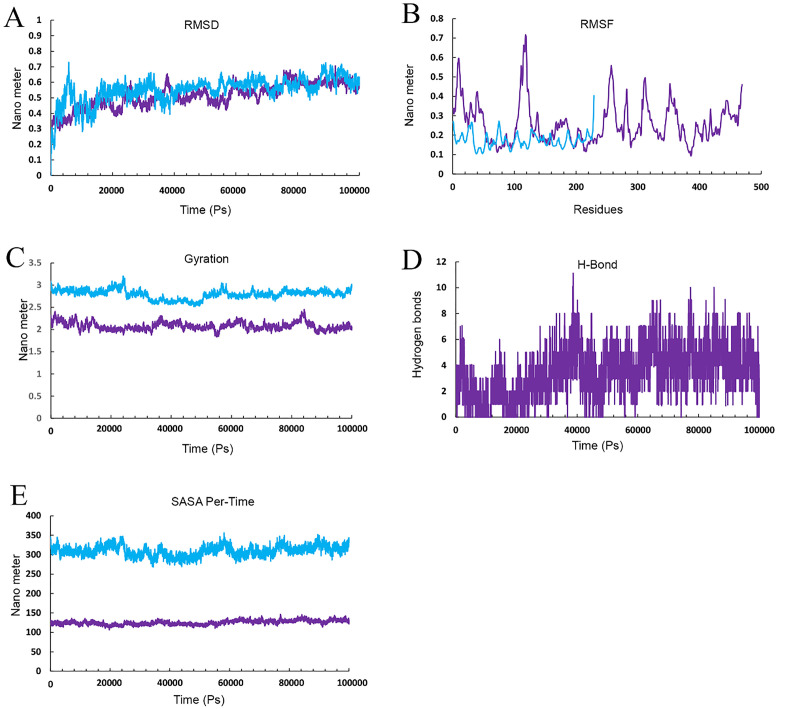
Molecular dynamics (MD) simulation results for the Cov19T-Heavy chain complex, evaluated over a 100 ns trajectory using GROMACS to assess structural stability and interaction dynamics. (A) Root Mean Square Deviation (RMSD) plot, showing the backbone deviation of Cov19T (purple) and Heavy chain (blue) over time. Cov19T stabilizes at 0.5 nm after 10 ns, similarity the Heavy chain reaches equilibrium at 0.5 nm after 10 ns, indicating stable complex formation. (B) Root Mean Square Fluctuation (RMSF) plot, depicting residue-level flexibility. Cov19T exhibits low fluctuations 0.32 in epitope regions, while the Heavy chain shows higher fluctuations 0.18 nm in non-binding regions, suggesting conformational flexibility. (C) Radius of Gyration (Rg) plot, measuring the compactness of the complex. Both Cov19T and Heavy chain maintain stable Rg values 3 nm for Cov19T and 2.2 for Heavy chain, confirming structural integrity throughout the simulation. (D) Hydrogen bond (H-Bond) analysis, illustrating the number of H-bonds between Cov19T and Heavy chain over time. An average of 2–6 H-bonds is maintained, highlighting strong intermolecular interactions. (E) Solvent Accessible Surface Area (SASA) per-time plot, showing the solvent exposure of Cov19T (purple) and Heavy chain (blue). SASA values stabilize at 320 nm for Cov19T and 130 nm² for Heavy chain, indicating minimal unfolding and consistent surface accessibility for immune recognition. These results collectively demonstrate the stability and interaction dynamics of the Cov19T-Heavy chain complex, supporting its potential for effective immune response elicitation.

**Fig 13:**
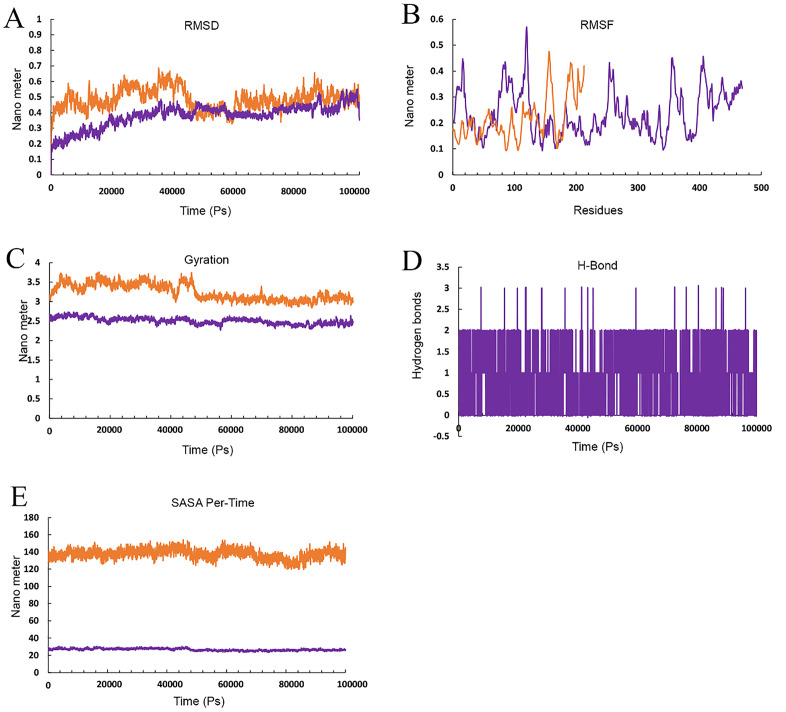
Molecular dynamics (MD) simulation results for the Cov19T-Light chain complex, evaluated over a 100 ns trajectory using GROMACS to assess structural stability and interaction dynamics. (A) Root Mean Square Deviation (RMSD) plot, showing the backbone deviation of Cov19T (purple) and Light chain (copper) over time. Cov19T stabilizes at 0.4 nm after 50 ns, similarity the Light chain reaches equilibrium at 0.4 nm after 50 ns, indicating stable complex formation. (B) Root Mean Square Fluctuation (RMSF) plot, depicting residue-level flexibility. Cov19T exhibits low fluctuations 0.30 in epitope regions, while the Light chain shows higher fluctuations 0.25 nm in non-binding regions, suggesting conformational flexibility. (C) Radius of Gyration (Rg) plot, measuring the compactness of the complex. Both Cov19T and Light chain maintain stable Rg values 3.5 nm for Cov19T and 2.5 for Light chain, confirming structural integrity throughout the simulation. (D) Hydrogen bond (H-Bond) analysis, illustrating the number of H-bonds between Cov19T and Light chain over time. An average of 0–2 H-bonds is maintained, highlighting strong intermolecular interactions. (E) Solvent Accessible Surface Area (SASA) per-time plot, showing the solvent exposure of Cov19T (purple) and Light chain (copper). SASA values stabilize at 140 nm for Cov19T and 13 nm² for Light chain, indicating minimal unfolding and consistent surface accessibility for immune recognition. These results collectively demonstrate the stability and interaction dynamics of the Cov19T- Light chain complex, supporting its potential for effective immune response elicitation.

Results from the MD simulation revealed that in the Cov19T-Heavy chain complex and Cov19T-Light chain complex, the hydrogen bond was established with a RMSD of 0.5 and 0.6 nm, respectively (Fig 12A and 13A). The RMSF diagrams for the residues in the Cov19T-Heavy chain complex and Cov19T-Light chain complex during the MD simulation showed minimal fluctuations (Fig 12B and 13B).

In the radius of gyration plots for both the Cov19T-Heavy chain and Cov19T-Light chain complexes, Cov19T exhibited greater stability than the Heavy chain and Light chain (Fig 12C and 13C). Additionally, Fig 12D and Fig 13D show hydrogen bonds number in the Cov19T- Heavy chain complex was higher than in the Cov19T-Light chain complex.

The per-time specific solvent-accessible surface area (SASA) graph indicated that Cov19T maintained a more stable structure compared to the Cov19T-Heavy chain complex and Cov19T-Light chain complex (Fig 12E and 13E). These results suggest that Cov19B remained relatively stable during binding with the Heavy chain. Notably, the Cov19T- Heavy chain complex and Cov19T-Light chain complex show its stability after 10 ns, in comparison the Cov19B-Heavy chain complex and Cov19T-Light chain complex show its stability after 5 ns.

MM/PBSA calculations confirmed the binding affinities of all complexes. Table 8 displays the computed energy of the bonds formed in each complex between the vaccines and Abs. The overall binding energy of the Cov19B-Heavy chain complex was −35.77, indicating a high affinity between the two proteins. The Cov19B-Light chain complex showed binding energy of −39.24.

**Table 8:** MMP/BSA analysis data.

Frames	VDWAALS	EEL	EPB	ENPOLAR	GGAS	GSOLV	TOTAL
**Complex**	**Cov19B-Heavy chain**						
**Average**	−53.35	−117.63	140.91	−5.7	−170.98	135.21	−35.77
**SD**	4.4	13.91	13.34	0.29	13.94	13.21	5.59
**SEM**	0.62	1.95	1.87	0.04	1.95	1.85	0.78
**Complex**	**Cov19B-Light chain**						
**Average**	−54.98	−159.08	180.64	−5.81	−214.07	174.83	−39.24
**SD**	4.39	22.25	21.69	0.18	22.6	21.68	7.52
**SEM**	0.62	3.12	3.04	0.02	3.16	3.04	1.05
**Complex**	**Cov19T-Heavy chain**						
**Average**	−24.98	−8.79	30.81	−2.95	−33.77	27.86	−5.91
**SD**	2	5.36	5.17	0.15	5.95	5.12	6.4
**SEM**	0.28	0.75	0.72	0.02	0.83	0.72	0.9
**Complex**	**Cov19T-Light chain**						
**Average**	−27.98	57.17	−45.81	−2.63	29.18	−48.44	−19.26
**SD**	2.46	10.52	10.09	0.14	10.65	10.11	2.49
**SEM**	0.34	1.47	1.41	0.02	1.49	1.42	0.35

The Cov19T-Heavy chain complex exhibited a binding energy of −5.91. The presence of a salt bridge between the Cov19T and Heavy chain structures may explain the less negative binding energy observed in the Cov19T-Heavy chain complex. Meanwhile, the Cov19T-Light chain complex showed a binding energy of −19.26. These values suggest that the structures of Cov19B and Cov19T bind effectively to mAb chains.

**Fig 14:**
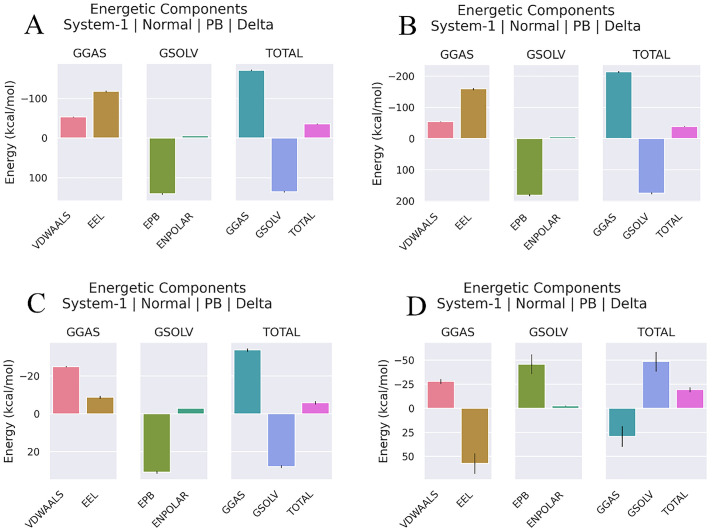
MMPBSA analysis of binding free energy contributions for the complexes, derived from 100 ns molecular dynamics simulations using GROMACS. The plot illustrates the decomposition of binding energy into individual components, including van der Waals (vdW), electrostatic, polar solvation, and non-polar solvation energies for (A) Cov19B-Heavy chain, (B) Cov19B-Light chain, (C) Cov19T-Heavy chain, and (D) Cov19T-Light chain complexes. The total binding free energy for the complex’s interaction is calculated, with significant contributions from van der Waals interactions and electrostatic interactions, partially offset by polar solvation energy. The non-polar solvation energy contributes favorably, enhancing the stability of the complex. These energy contributions highlight the strong affinity between complexes, supporting the potential of structures to elicit a robust immune response through stable receptor binding, a critical factor in its efficacy as a multi-epitope vaccine candidate against SARS-CoV-2 variants.

Subsequent studies reveal that other energies have a significant impact on the complexes under study, in addition to the total binding energy. To be more precise, the electrostatic energy is recorded at −117.63 and the van der Waals energy contribution is −53.35 in the Cov19B-Heavy chain complex. These values highlight how the complex’s molecular forces interact intricately, providing insight into the complex dynamics that govern the complex’s stability and interactions (Fig 14A). In the Cov19B-Light chain complex, the van der Waals energy is −54.98, and the electrostatic energy is −159.08 (Fig 14B).

These values provide insight into the specific forces driving the interactions within the Cov19B-Heavy chain and Cov19B-Light chain complexes.

The electrostatic energy in the Cov19T-Heavy chain complex is −8.79 and the van der Waals energy is −24.98, as shown in Fig 14C. On the other hand, as Fig 1D illustrates, the van der Waals energy in the Cov19T-Light chain complex is −27.98, and the electrostatic energy is 57.17. All of these quantitative evaluations indicate that both complexes are stable, with particular attention paid to the strong stability shown in Cov19B-Heavy chain, Cov19B-Light chain, and Cov19T-Heavy chain complexes. These findings highlight the complex intermolecular force balance that controls the formation of complexes and offer valuable new insight into their structural integrity and possible functional implications.

### Immune simulation

**Fig 15:**
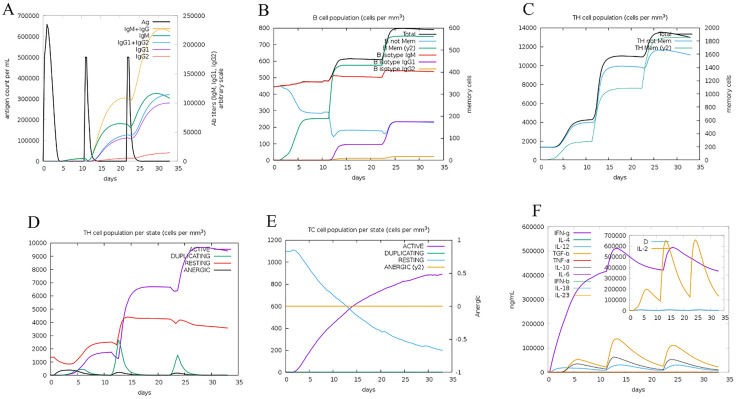
C-ImmSim simulation results of the immunological response induced by the multi-epitope vaccine construct Cov19-B, evaluated following a three-dose regimen (injections at 0, 4, and 8 weeks) to assess its efficacy against SARS-CoV-2. (A) Levels of immunoglobulins (IgM, IgG1, IgG2) over time, showing a significant increase post-vaccination, with peak IgG levels reaching after the third dose, indicating a robust humoral immune response. (B) Total B-cell isotype populations, demonstrating a shift towards IgG1 and IgG2 isotypes, reflecting effective class-switching and antibody production. (C) Population dynamics of CD4 T-helper cells, which peak, following the second dose, highlighting the vaccine’s ability to stimulate cellular immunity. (D) Memory and total T-helper cell counts, with memory T-helper cells increasing to by week 12, ensuring long-term immune protection. (E) Population of CD8 T-cytotoxic lymphocytes, showing a sustained increase, after the third dose, indicative of strong cytotoxic activity against infected cells. (F) Production levels of cytokines IL-4, IL-6, and IL-12 over time, with IL-12 peaking, promoting Th1 differentiation, while IL-4 and IL-6 support Th2 responses and B-cell activation. These results collectively demonstrate that Cov19-B elicits a balanced humoral and cellular immune response, supporting its potential as an effective vaccine candidate against SARS-CoV-2 variants.

**Fig 16:**
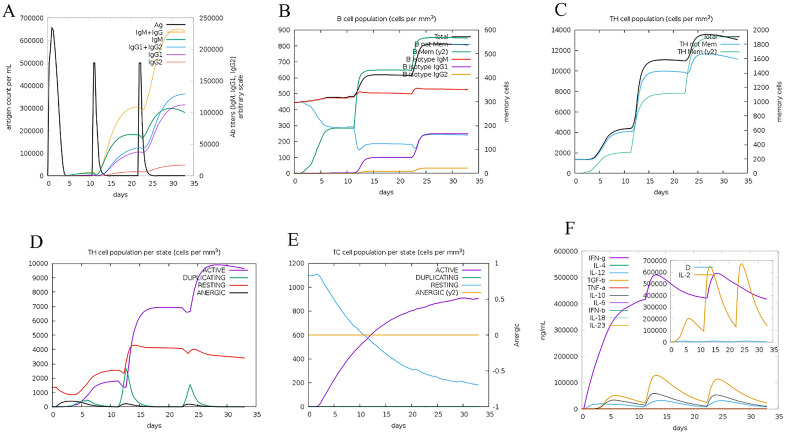
C-ImmSim simulation results of the immunological response induced by the multi-epitope vaccine construct Cov19-T, evaluated following a three-dose regimen (injections at 0, 4, and 8 weeks) to assess its efficacy against SARS-CoV-2. (A) Levels of immunoglobulins (IgM, IgG1, IgG2) over time, showing a significant increase post-vaccination, with peak IgG levels reaching after the third dose, indicating a robust humoral immune response. (B) Total B-cell isotype populations, demonstrating a shift towards IgG1 and IgG2 isotypes, reflecting effective class-switching and antibody production. (C) Population dynamics of CD4 T-helper cells, which peak, following the second dose, highlighting the vaccine’s ability to stimulate cellular immunity. (D) Memory and total T-helper cell counts, with memory T-helper cells increasing to by week 12, ensuring long-term immune protection. (E) Population of CD8 T-cytotoxic lymphocytes, showing a sustained increase, after the third dose, indicative of strong cytotoxic activity against infected cells. (F) Production levels of cytokines IL-4, IL-6, and IL-12 over time, with IL-12 peaking, promoting Th1 differentiation, while IL-4 and IL-6 support Th2 responses and B-cell activation. These results collectively demonstrate that Cov19-T elicits a balanced humoral and cellular immune response, supporting its potential as an effective vaccine candidate against SARS-CoV-2 variants.

The C-IMMSIM immunological study provided an *in-silico* perspective on the vaccine’s effectiveness and yielded valuable insights into the immunogenic profile the vaccine elicited. Interestingly, compared to the secondary and tertiary immune responses, the primary immune response was shown to occur at substantially lower levels after stimulation. Based on these results, it was calculated that to adequately boost the immune system’s response, a vaccination program consisting of three doses spaced four weeks apart would be required. The resulting immune responses showed IgM and IgG levels (Fig 15A and 16A).

One noteworthy finding in the tertiary response was the appearance of memory cells, which are indicative of a critical component of long-term immune defense. As seen in Figs 15B and 16B, this phase was marked by a robust activation of B cells, including B isotypes of IgM and IgG. At the same time, as Figs 15C and 16C show, there was a notable increase in the activity of CD4 T-helper cells and CD8 cytotoxic T lymphocytes, which are associated with secondary and tertiary responses. Nevertheless, it is significant that this increased activity eventually decreased. Furthermore, there was an association between the cytotoxic T cell (TC) population and the pre-activation of all TCs, suggesting that the immune system was responding in a coordinated and anticipatory manner (Fig 15D and 16D).

The final results of the investigation showed increased concentrations of key cytokines as well as significant effects on populations of CD8 T-cytotoxic lymphocytes after the injections. This finding highlights the vaccine’s ability to effectively activate the immune system, as Figs 15E, 16E, 15F, and 16F illustrate. All of these results point to the vaccine’s potential efficacy in stimulating a robust and diverse immune response. Ultimately, this analysis’s *in-silico* immune response predictions offer valuable insights into the vaccine’s anticipated effectiveness, contributing to well-informed decisions on its distribution and refinement.

## Discussion

Numerous studies on SARS-CoV-2 provide a wealth of information, including the origin of the virus (bats), epidemiology, clinical features, and treatment strategies including antiviral drugs (remdesivir and nimratrelivir/ritonavir), monoclonal antibodies, and vaccines (BNT162b2 and mRNA-1273). It also addresses the evolution of the virus, mutations, and challenges associated with vaccine and therapeutic efficacy, emphasizing the importance of vaccination and public health measures [45,48,56–62].

SARS-CoV-2, as a global challenge, requires multifaceted approaches including widespread vaccination, development of effective treatments, and international cooperation. Despite significant advances in vaccine and antiviral drug development, ongoing virus mutations and declining vaccine efficacy over time highlight the need for continued surveillance, enhanced vaccine doses, and implementation of public health interventions. These findings underscore the need for adaptive strategies to control the pandemic and mitigate its impacts [63–74].

The rapid evolution of SARS-CoV-2 variants, such as Delta and Omicron, poses a significant challenge due to immune evasion and reduced efficacy of existing vaccines and therapeutics [42]. The immunopathogenesis of COVID-19, driven by dysregulated TLR-mediated cytokine storms and impaired adaptive immunity, underscores the need for vaccines that elicit robust, broad-spectrum protection [38,40]. This study addresses this problem by designing two multi-epitope vaccine constructs, Cov19-B and Cov19-T, targeting conserved epitopes across SARS-CoV-2 proteins to ensure coverage against variants. The necessity of this approach is evident given the limitations of current therapeutics, such as antivirals and monoclonal antibodies, which struggle against emerging variants [42].

Immunoinformatics enabled precise epitope selection using IEDB, NetCTL, Propred, and ABCpred, ensuring antigenicity, safety, and conservation [43,75]. The use of β-defensin and PADRE adjuvants, EAAAK and GPGPG linkers, and disulfide engineering enhanced immunogenicity and stability, as validated by molecular docking, dynamics simulations, and C-ImmSim immune simulations predicting strong IgG, IFN-γ, and IL-2 responses [44]. Immunoinformatics has proven instrumental in vaccine design against diverse pathogens, including viruses, bacteria, and helminths like Schistosoma mansoni and Fasciola hepatica. By identifying conserved epitopes and simulating immune responses, it offers a rapid, cost-effective approach to combat complex parasitic lifecycles and antigenic variability [76–80].

While this study is limited to in silico predictions, the methodologies align with validated approaches for SARS-CoV-2 and other pathogens [37]. Future experimental validation, such as in vitro binding assays or animal models, is needed to confirm efficacy, as demonstrated in similar studies [46]. Cov19-B and Cov19-T represent promising candidates for broad-spectrum SARS-CoV-2 vaccination, with potential adaptability to co-infections in endemic regions [43].

After the discovery of SARS-CoV-2 and the declaration of COVID-19 as a pandemic by the World Health Organization, scientists initiated efforts to develop vaccines aimed at mitigating the impact and transmission of the disease [81]. Vaccines that are live or attenuated have a long history of efficacy, but they can trigger autoimmune and allergic reactions. As a result, immunoinformatics technologies are now being used to eliminate biosafety concerns while also saving time and money. Multi-epitope vaccines have the potential to overcome the limitations of traditional vaccines. One of the most notable differences between multi-epitope vaccines and traditional vaccines is that personalized immune responses are elicited following proper identification of immunogenic epitopes, enabled by viral genome screening. Several SARS-CoV-2 vaccines have been developed and reported to date [33,82–85].

The gradual emergence of different variants reflects the evolutionary process of mutation, selection, and adaptation in the severe acute respiratory syndrome coronavirus 2 (SARS-CoV-2). Modifications in the amino acid composition of the spike protein lead to changes in the virus’s antigenic properties, transmissibility, and pathogenicity [86].

In general, the occurrence of mutations in the structure of SARS-CoV-2 enables it to escape

the immune system’s defenses or evade immune responses [87]. These mutations, in turn, can lead to the emergence of new variants that give SARS-CoV-2 the ability to bypass immune defense mechanisms and ultimately reduce the effectiveness of vaccines [88,89]. Therefore, the study, identification and application of these mutations can lead to the development of mutation-proof vaccines to combat COVID-19, which may be capable of acting against a wider range of COVID-19 variants [87].

The current study was carried out with the aim of addressing the lack of identified mutations in the spike structure and incorporating alternative amino acids in the epitopes bearing these mutations. It is expected that the mutations used in the structures will facilitate the design and prediction of a mutation-proof vaccine against SARS-COV-2.

The S-protein of these viral strains has undergone many mutations, which have rendered vaccines ineffective [89]. The frequent instances of recombination pose a challenge in predicting the efficacy of vaccines targeting the spike protein. Recombination has the potential to confer modified transmissibility, virulence, and immune escape properties on the virus [90,91]. Evidence has shown that in the S protein, the highest mutation frequency was observed in amino acids (aa) 508–635 (0.77%) and aa 381–508 (0.43%) [92].

One report has demonstrated the dominance of the SARS-CoV-2 variant with the D614G Spike mutation [93]. The D614 variant was prevalent in epidemics when G614 began to appear, and the G614 variant exhibits a higher titer as pseudotyped virions. The D614G mutation is part of a conserved haplotype with four mutations that consistently occur together. The increasing frequency of G614 in populations co-circulating with D614 suggests positive selection. Additionally, G614 is associated with higher viral nucleic acid levels in the upper respiratory tract and increased infectivity in pseudotyping assays [93]. Furthermore, the G614 variant in the Spike protein has spread more rapidly than the D614 variant globally, suggesting increased infectivity. This finding aligns with in vitro observations of higher infectivity with G614 Spike-pseudotyped viruses and the G614 variant’s association with lower patient Ct values, indicating potentially higher in vivo viral loads [93].

The SARS-CoV-2 variant carrying the G614 mutation has become the predominant circulating variant, replacing the D614 variant. The D614G substitution disrupts a hydrogen bond interaction, leading to the destabilization of the spike trimer and an increased interaction of the receptor binding domain (RBD) with the angiotensin-converting enzyme 2 (ACE2). This mutation, by elevating the viral load in the upper respiratory tract of COVID-19 patients, is suggested to enhance the transmission of SARS-CoV-2 [94,95].

Nevertheless, in a report of D614G, Epsilon, Alpha, Beta, and Gamma mutations found in pan-sarbecovirus vaccine candidate (DCFHP-alum) were associated with a decreased efficacy of DCFHP-alum [96].

Notably, it has been shown that certain substitutions in the spike protein, such as Q493R and Q498R, were consistent with the B.1.1.529 (Omicron) variant, while others like Y144del and H655Y were also identified in B.1.1.7 (Alpha), P.1 (Gamma), and B.1.1.529 (Omicron) variants [97–100]. On the other hand, it has been noted that the similarly located alteration P681R increases the Delta variant’s pathogenicity and ability to replicate. The virus’s antigenic epitope is gradually modified by these mutations as they accumulate one after the other. As a result of this process, “genetic drift” gives way to “antigenic drift “ [101–103].

There is even a report that shows that three key amino acid mutations in the S protein, including A605E, E633Q, and R891G, increase the infection efficiency of recombinant viruses in the Coronavirus porcine epidemic diarrhea virus (PEDV) to Infect Vero cells [104].

In the case of the Beta variant (B.1.351), alterations such as mutations at positions 242–244, K417N, E484K, and N501Y have demonstrated notable resistance against infection or vaccine-induced neutralizing antibodies [105–109]. The concurrent presence of E484K and N501Y mutations synergistically boosts the spike protein’s affinity for the human ACE2 receptors [110,111].

The Delta variant, also known as B.1.617.2, harbors multiple mutations that have been observed in other Variants of Concern. These mutations, including L452R, T478K, E484Q, D614G, and P681R, are all situated within the spike protein [103]. The L452R mutation has been linked to higher viral fusogenicity, decreased sensitivity to neutralizing antibodies, and increased infectivity. All of these factors work together to increase the virus’s reproductive rate [112].

The development of SARS-CoV-2 prior to the Omicron variants was mainly driven by the accumulation of recurrent mutations in important spike protein residues, including D614, N501, P681, K417, and E484. However, this evolutionary trajectory underwent a major change with the emergence of the Omicron variant and associated sublineages [86].

The sublineage BA.4.6, which descended from the BA.4 variant, is unique in that it carries two additional mutations located in the spike protein. These mutations, designated R346T and N658S, are changes that set BA.4.6 apart from its progenitor strain, BA.4 [113,114]. Also, the BF.7 variants, derived from BA.5, include an additional R346T mutation. This specific mutation has been linked to an increased capability of the virus to evade neutralizing antibodies produced by vaccines or prior infections [115–117]. Ma *et al*. (2023) reported an increase in Sarbecovirus fusogenicity by enhancing the usage of TMPRSS2 through the Spike substitution T813S [118].

Similar to our study, Bhattacharya *et al.* (2023) used the predicted mutations of Gly339Asp, Asn501Tyr, Ser477Asn, Thr478Lys, Tyr505His on SARS-CO2 spike to build the final structure of their proposed vaccine [89]. In addition, Zhang *et al.* (2022), used mutations such as Lys417Asn (Variants Of Concerns or VOCs in Delta), T478K (VOCs in Delta, Omicron), N501Y (VOCs in Alpha, Beta, Gamma, Omicron), E484K (VOCs in Beta, Gamma), K417T (VOCs in Gamma), S477N (VOCs in Omicron), K417N (VOCs in Beta, Omicron) and F490S (VOCs in Lambda) with the aim of fighting against different SARS-COV-2 variants in their construct [119].

Although the mutation data in this study were collected up to January 2022, they represent a substantial and diverse set of SARS-CoV-2 sequences including several high-frequency mutations that persist in later variants. Importantly, our methodology is adaptable and can be readily updated to include novel mutations as new sequence data emerge. Future iterations of the vaccine constructs can thus integrate real-time mutational trends, ensuring continued relevance against evolving strains

A variety of spike mutations and amino acid substitutions are listed in Table 9, where all mutations on epitopes containing amino acid substitutions with high antigenicity are mentioned and confirmed with other studies.

**Table 9:** Uniqueness of the selected epitopes carrying notable mutation on spike SARS-COV-2 to worldwide.

Start	Allele	Peptide	Mutation/s	Reference of studied mutants	Reference of used mutants in vaccine
13	B-Cell	SQCVNLTTRTQLPPAYTNSFTRGVY	Leu18Phe, Pro26Ser, Thr19Arg, Thr19Ile,	[120–122]	-
59	B-Cell	FSNVTWFHAIHASGTNGTKRFDN	Ala67Val	[123]	-
138	B-Cell	DPFLGVYYHKNNKSWME	Asp138Tyr, Gly142Asp, Tyr145His	[121,123,124]	-
206	B-Cell	KHTPINLVRDLPQGFS	Val213Gly	[125]	-
329	B-Cell	FPNITNLCPFGEVFNATRFASVYAWNRKRISNCVA	Gly339Asp, Arg346Lys, Arg346Thr	[122,126,127]	Gly339Asp [89]
369	B-Cell	YNSASFSTFKCYGVSPTKLNDLCFT	Ser371Pro, Ser371Phe, Ser373Pro, Ser375Phe, Thr376Ala*	[128,129]	-
404	B-Cell	GDEVRQIAPGQTGKIADYNYKLP	Asp405Asn, Arg408Ser, Ile410Met*, Lys417Asn	[130–133]	Lys417Asn [119]
440	B-Cell	NLDSKVGGNYNYLYRLFRKSNLKPFERDISTEIYQAGSTPCNGVEGFNCYFPLQSYGFQPTN	Asn440Lys, Gly446Ser, Leu452Gln, Leu452Arg, Ser477Asn, Thr478Lys, Phe486Val, Glu484Ala, Gln493Arg, Gly496Ser, Gln498Arg, Asn501Tyr	[112,121,123,134–142]	Asn501Tyr, Ser477Asn, Thr478Lys, Glu484Ala [89,119,143]
656	B-Cell	HVNNSYECDIPI	His655Tyr	[144]	-
672	B-Cell	ASYQTQTNSPRRARSVASQ	Pro681His	[145,146]	-
695	B-Cell	YTMSLGAENSVAYSNN	Ala701Val	[142]	-
786	B-Cell	KQIYKTPPIKDFGGF	Asp796Tyr*		-
1107	B-Cell	RNFYEPQIITTD	Asp1118His	[123,142]	-
1133	B-Cell	VNNTVYDPLQPELDSFKEELDKYFKNHTSPDVDLGDISGI	Asp1146Glu*		-
15	HLA-B1402	VN **LRTRTQL**	Leu18Phe, Pro26Ser, Thr19Arg, Thr19Ile,	[120–122]	-
17	HLA-B7301	**LRTRTQLPP**	Leu18Phe, Pro26Ser, Thr19Arg, Thr19Ile,	[120–122]	-
18	HLA-A3001	**RTRTQLPPA**	Leu18Phe, Pro26Ser, Thr19Arg, Thr19Ile,	[120–122]	-
19	HLA-C0702	**TRTQLPPA** Y	Leu18Phe, Pro26Ser, Thr19Arg, Thr19Ile,	[120-122]	-
92	HLA-C0303, HLA-C0802, HLA-C1203	FASTEKSNI	Thr95Ile	[147]	-
482	HLA-C0401	GFNCYFPLQ	Glu484Ala	[138, 148]	-
492	HLA-A6601, HLA-A6901, HLA-C1203	YGFQPTN **GV**	Gly496Ser	[139]	-
503	HLA-C1203	**VGYQPYRVV**	Tyr505His	[149,150]	-
568	HLA-A2501, HLA-A6901, HLA-A6802	DIADTADAV	Ala570Asp	[151]	-
612	HLA-A0250, HLA-A0206, HLA-A0212	YQ**DVNCTEV**	Asp614Gly	[152]	Asp614Gly [153]
614	HLA-A0216, HLA-A0212	**DVNCTEV**PV	Asp614Gly	[152]	Asp614Gly [153]
678	HLA-B0802, HLA-B0801, HLA-B1402, HLA-C1502	N **SPRRARSV**	Pro681Arg	[146]	-
679	HLA-B1402, HLA-B7301	**SPRRARSVA**	Pro681Arg	[146]	-
680	HLA-B0803, HLA-B1402	**PRRARSVA** S	Pro681Arg	[146]	-
5	DRB3_0301, DRB4_0101, HLA-DQA10102-DQB10501	LV**LLPLVSSQCVNLT**	Leu5Phe, Val11Val, Leu18Phe, Thr19Arg, Thr19Ile	[120-122,128]	-
7	DRB3_0301, DRB4_0101, HLA-DQA10102-DQB10501	**LLPLVSSQCVNLTTR**	Val11Val, Leu18Phe, Thr19Arg, Thr19Ile	[120-122]	-
374	HLA-DQA10501-DQB10402, HLA-DQA10601-DQB10402, HLA-DQA10201-DQB10402	**FSTFKCYGVSPTKLN**	Ser375Phe, Thr376Ala*	[128,129]	-
375	HLA-DQA10501-DQB10402, HLA-DQA10201-DQB10402	**STFKCYGVSPTKLND**	Ser375Phe, Thr376Ala	[128,129]	-
502	HLA-DQA10501-DQB10402, HLA-DQA10303-DQB10402	G**VGYQPYRVV**VLSFE	Tyr505His	[149,150]	Tyr505His [89]
503	HLA-DQA10303-DQB10402, HLA-DQA10501-DQB10402	**VGYQPYRVV**VLSFEL	Tyr505His	[149,150]	Tyr505His [89]
504	HLA-DPA10103-DPB10601	**GYQPYRVV**VLSFELL	Tyr505His	[149,150]	Tyr505His [89]
537	DRB1_0901	K**CVNFNFNGLTGTGV**	Thr547Lys	[139]	-
538	DRB1_0901	**CVNFNFNGLTGTGVL**	Thr547Lys	[139]	-
539	DRB1_0101, DRB1_0901,	**VNFNFNGLTGTGVLT**	Thr547Lys	[139]	-
540	DRB1_0101, DRB1_0901	**NFNFNGLTGTGVLT**E	Thr547Lys	[139]	-
715	HLA-DQA10601-DQB10402, HLA-DQA10201-DQB10303, DRB1_0701, DRB1_0901, HLA-DQA10201-DQB10202, HLA-DQA10201-DQB10402	**PTNFTISVTTEILPV**	Thr716Ile	[123]	-
716	DRB1_0701, DRB1_0901, HLA-DQA10201-DQB10202, HLA-DQA10201-DQB10301	**TNFTISVTTEILPVS**	Thr716Ile	[123]	-

* So far, they have only been seen in the GISAID database.

We designed two constructs capable of triggering both humoral and cellular immune responses a combination of computational tools and immunoinformatics methodologies in our research. Here, we harnessed a multi-epitope vaccination against the Spike protein, as well as mutations in the variants of Spike protein, in this study [85,153].

As an adjuvant, we employed beta-defensin 3 sequences. The C-terminal region of Cov19B has been found to behave as a possible adjuvant when combined with beta-defensin 3. We also inserted the PADRE sequence, which serves as a peptide with excellent adjuvant characteristics, into construct.

Additionally, we used the Large ribosomal subunit protein bL12, 50S ribosomal protein L7/L12 as a potent protein adjuvant for Cov19T structure. The GPGPG linkers allowing neighboring domains to operate more freely; it also has immune-modulatory properties [154]. Antigen processing was also been aided by the KK linker.

The selection of β-defensin 3 and PADRE as adjuvants for the Cov19-B and Cov19-T constructs, respectively, was driven by their complementary immunological roles. β-defensin 3, a natural antimicrobial peptide, enhances innate immunity by activating TLR4 on antigen-presenting cells, thereby promoting B-cell responses critical for neutralizing SARS-CoV-2 [45]. HBD-3 activates innate immunity by enhancing antigen uptake and APC maturation. Its known antimicrobial and immunomodulatory properties make it a valuable front-line enhancer of host defense.

Conversely, PADRE, a synthetic universal T-helper epitope, ensures robust CD4 + T-cell activation across diverse MHC class II alleles, enhancing cytotoxic T-cell responses against viral variants. PADRE provides a broad T-helper response across diverse HLA backgrounds, improving CD4 + T cell recruitment regardless of host genotype [57]. The EAAAK and GPGPG linkers were chosen to optimize the structural and functional integrity of the vaccine constructs. EAAAK provides rigidity and separates functional domains (adjuvants from epitope blocks) to prevent unfavorable interactions. EAAAK provides rigidity and separates functional domains (adjuvants from epitope blocks) to prevent unfavorable interactions. The rigid EAAAK linker prevents domain interference, while the flexible GPGPG linker facilitates epitope presentation to MHC molecules, as demonstrated in prior multi-epitope vaccine studies. GPGPG is a neutral, flexible linker ideal for MHC-II epitope separation, preserving their structure and immunogenicity [48]. KK enhances solubility and introduces structural flexibility, improving protein folding and expression. AAY facilitates proteasomal cleavage for effective MHC-I presentation. These choices align with the goal of designing a safe, immunogenic, and broadly protective vaccine against SARS-CoV-2 variants. Linkers were carefully chosen to preserve the immunological function of each epitope and reduce potential interference. This rational integration of adjuvants and linkers provides a structurally robust and immunologically potent construct that aims to elicit comprehensive immune protection.

The computed index identified specific epitopes that frequently bind to HLA molecules in target populations, providing coverage for more than 95% of the population’s cytotoxic T lymphocytes (CTLs) and helper T lymphocytes (HTLs) across 64 different geographic areas. A mix of linear and conformational B cell epitopes was added to the construct so that this specific epitope only elicits an immune response in those whose expressed MHC can attach to it. Peptides linked to cytotoxic T lymphocyte (CTL) responses require HLA I molecules, whereas peptides linked to helper T lymphocyte (HTL) responses require HLA II molecules. Thus, different HLAs can bind to the CLT and HTL epitopes.

MHS coverage is determined by calculating the percentage of HLA classes. Achieving a broader range of HLA molecule binding for each epitope contributes to significant population coverage among related individuals [155].

Seventy-three CTL epitopes and forty HTL epitopes were discovered from the spike, demonstrating the construct’s ability to produce high cellular immunity. We employed the AllerCatPro server to forecast probable allergenicity while paying attention to the significance of non-allergenicity for the immune system, which is often one of the critical inherent limitations in the vaccine design phase. The findings indicated that none of the epitopes tested were immune system allergens. Furthermore, toxicity testing of selected epitopes revealed that each epitope was expected to be non-toxic.

To determine the third protein’s structure, the I-TASSER server was employed, followed by refinement using GalaxyRefine. The structures showed improvement post-refinement, which is evident in comparing Z-scores before and after the refining process. Subsequently, the 3D-modeled construct’s concerning the amino acid sequence position revealed a high proportion of residual negative energy and a low proportion. These findings indicate potential defects or inaccuracies, which contributed minimally to the structural energy plot.

Validation related to homology modeling association suggests that most of the structure possesses favorable energy levels, contributing to overall stability. In contrast regions with higher energy are relatively limited in the construct, indicating the structure’s low energy and thus its strength in terms of energetic content. Proteins with stable structures can better maintain connections with other proteins and ligands. Furthermore, following refining, the Ramachandran graph revealed that 85% (Cov19B) and 72% (Cov19T) of amino acids have appropriate locations.

Pattern recognition receptors (PRR) recognize pathogen-associated molecular patterns (PAMPs) and activate the responsive innate host antiviral defense, which aids in the regulation of viral infection [156]. Since COVID-19 is a relatively new disease with limited clinical evidence, research has revealed that immune stimulation is essential in COVID-19 vaccinations.

The interaction of the recombinant protein of this vaccine with mAb was investigated using protein–protein molecular docking. The interactions generated in both CoV19B-Heavy chain, CoV19B-Light chain, CoV19T-Heavy chain, and CoV19T-Light chain complexes were explained by hydrogen bonds in CoV19B-Heavy/Light chain and CoV19-Heavy/Light chain complexes. The interaction of complexes showed a high affinity for them. Other binding energies indicate a strong affinity for the complexes [157].

Here, free energy calculations and molecular dynamics simulations were used to assess and simulate the vaccine model’s docking with monoclonal antibodies. Research has suggested a connection between heavy chain triggering and light chain signaling [158]. It can then activate immunological pathways implicated in pathogenesis and viral infection as a result [159–161]. Consequently, we conducted docking of the vaccine construct with the monoclonal antibody (mAb), and the MD complex data in the CoV-mAb system indicated appropriate interaction between the heavy chain/light chain and the CoV19B/T.

Studies show that neutralizing plasmas from donors who have recovered from COVID-19 exhibit a variety of antibody responses [162,163]. In one report, it has been examined polyclonal plasma IgGs, which exhibited different levels of cross-reactivity against the RBDs of the common cold virus and non-cross-reactive antibodies against S proteins from SARS-CoV-2, SARS-CoV, and MERS-CoV. Convalescent plasma IgG epitope mapping revealed an S1A epitope outside the RBD, which may serve as an alternate binding site for neutralizing antibodies, in addition to the expected targeting of the S protein RBD [162]. Also, examination of a neutralizing monoclonal antibody (mAb) from different patients revealed the presence of an anti-SARS-CoV-2 antibody that blocks the ACE2 receptor [19,162].

The idea that recurrent groups of anti-SARS-CoV-2 neutralizing antibodies from the VH gene segments use different RBD-binding mechanisms, as observed in several mAbs, respectively, is supported by structural findings in other research. This data is essential for analyzing the sequences of antibodies produced by vaccination or infection. Notably, frequent changes in different SARS-CoV-2 isolates seem unlikely to disrupt the identified RBD and S1A epitopes, providing hope for the efficacy of vaccines against the ongoing epidemic.

Research has shown that some antibodies potently neutralize the virus, correlating with their ability to compete with ACE2 for RBD binding. These antibodies and infected plasma did not cross-react with RBDs of SARS-CoV or MERS-CoV, although cross-reactivity was observed with their trimeric spike proteins. Crystal structure analysis has revealed that steric hindrance inhibiting viral engagement with ACE2, suggesting that anti-RBD antibodies are mainly viral-species-specific inhibitors [164].

In molecular dynamic simulations lasting 100 nanoseconds, the RMSD and RMSF diagrams exhibited minimal oscillations and fluctuations within the complexes, affirming the robust stability and flexibility of the vaccine structure. The RMSD, RMSF, and radius of gyration diagrams all indicated that the number of hydrogen bonds engaged in the complexes was maintained, which was supported by the amount of H-bonds.

## Conclusion

The simultaneous presence of various SARS-CoV-2 variants and co-infections in immunocompromised individuals accelerates the generation of recombinant forms. Monitoring genomic variations in SARS-CoV-2, with a focus on mutations in the spike protein and instances of recombination, is crucial. This surveillance allows for the ongoing detection of shifts in both the viral genome and antigenic epitopes, fostering the development of pioneering vaccination methods and treatment modalities. Here, we introduced notable mutations in the spike protein of the coronavirus and applied them to pan-sarbecovirus constructs against the spike of the coronavirus. The results show that despite the amino acid substitutions, there is a proper connection between the vaccine structure and the heavy and light chains of the human antibody. Based on the results and analysis of this study, these structures probably have the ability to elicit an appropriate immune response in the body, and the results show that these structures deserve further investigation in *in-vitro* and *in-vivo* experiments.

## Supporting information

S1 Table Population coverage calculation. (PDF)

S2 Table Predicted discontinuous epitope(s) of Cov19B. (PDF)

S3 Table Predicted discontinuous epitope of Cov19T. (PDF)

S4 Table Summary of the top 10 models for Cov19B-Heavy chain Table. (PDF)

S5 Table Summary of the top 10 models for Cov19B-Light chain Table. (PDF)

S6 Table Summary of the top 10 models for Cov19T-Heavy chain. (PDF)

S7 Table Summary of the top 10 models for Cov19T-Light chain. (PDF)

S1 Fig Results of Engineering disulfide for Cov19-B 3D structure. (PDF)

S2 Fig Results of Engineering disulfide for Cov19-T 3D structure. (PDF)
