# Supplementary material for: Designing a novel vaccine against COVID-19 based on spike SARS-Cov-2 notable mutations using immunoinformatics approaches
Source: PLoS One. 2026 Feb 26;21(2):e0334662. doi: 10.1371/journal.pone.0334662 (PMC12944808; doi:10.1371/journal.pone.0334662)
Supplement: S1 Table — (PDF) [file pone.0334662.s001.pdf]

Table S1. Population coverage calculation.

population/area	Class I	Class II	Class combined
	Coverage[a]		
Argentina	99.88%	98.62%	100.0%
Australia	93.67%	97.69%	99.85%
Austria	98.52%	98.14%	99.97%
Belgium	96.35%	98.09%	99.93%
Brazil	96.7%	99.89%	100.0%
Canada	0.0%	99.66%	99.66%
Central Africa	94.13%	98.28%	99.9%
Central America	9.26%	97.28%	97.53%
China	95.77%	99.33%	99.97%
Czech Republic	95.75%	99.75%	99.99%
Denmark	0.0%	98.42%	98.42%
East Africa	97.67%	98.92%	99.97%
England	99.23%	99.18%	99.99%
Europe	98.18%	99.96%	100.0%
Finland	97.82%	90.97%	99.8%
France	98.75%	99.99%	100.0%
Germany	98.71%	99.33%	99.99%
Greece	0.0%	98.24%	98.24%
Hong Kong	95.79%	0.0%	95.79%
India	91.31%	99.9%	99.99%
Indonesia	78.75%	99.01%	99.79%

Iran	96.24%	97.75%	99.92%
Ireland Northern	99.21%	98.63%	99.99%
Ireland South	99.35%	97.9%	99.99%
Israel	92.64%	98.92%	99.92%
Italy	97.65%	98.47%	99.96%
Japan	98.53%	98.94%	99.98%
Korea; South	97.7%	96.93%	99.93%
Malaysia	79.95%	93.15%	98.63%
Mexico	97.86%	99.89%	100.0%
Netherlands	0.0%	98.95%	98.95%
New Zealand	0.0%	99.03%	99.03%
North Africa	98.34%	99.05%	99.98%
North America	98.41%	99.99%	100.0%
Northeast Asia	95.78%	99.33%	99.97%
Norway	0.0%	99.21%	99.21%
Oceania	95.36%	99.21%	99.96%
Pakistan	93.61%	25.25%	95.22%
Philippines	94.08%	19.23%	95.22%
Poland	98.47%	99.09%	99.99%
Portugal	98.34%	99.19%	99.99%
Russia	97.48%	99.97%	100.0%
Saudi Arabia	98.36%	95.47%	99.93%
Scotland	38.2%	87.84%	92.48%
Singapore	92.9%	94.86%	99.63%
South Africa	98.51%	27.07%	98.91%

South America	97.15%	99.17%	99.98%
South Asia	93.74%	99.9%	99.99%
Southeast Asia	94.8%	97.43%	99.87%
Southwest Asia	94.75%	98.15%	99.9%
Spain	88.76%	99.97%	100.0%
Sweden	96.33%	99.98%	100.0%
Switzerland	7.84%	0.0%	7.84%
Taiwan	97.43%	98.68%	99.97%
Thailand	92.71%	97.88%	99.85%
Turkey	50.69%	96.44%	98.24%
Ukraine	0.0%	21.22%	21.22%
United Arab Emirates	4.55%	32.92%	35.97%
United Kingdom	14.07%	69.14%	73.48%
United States	98.45%	100.0%	100.0%
Vietnam	94.28%	94.74%	99.7%
Wales	1.39%	0.0%	1.39%
West Africa	98.15%	98.99%	99.98%
West Indies	98.54%	96.96%	99.96%
**Average**	**76.83**	**87.43**	**93.8**

[a] projected population coverage
