# Supplementary material for: Designing a novel vaccine against COVID-19 based on spike SARS-Cov-2 notable mutations using immunoinformatics approaches
Source: PLoS One. 2026 Feb 26;21(2):e0334662. doi: 10.1371/journal.pone.0334662 (PMC12944808; doi:10.1371/journal.pone.0334662)
Supplement: S2 Table — (PDF) [file pone.0334662.s002.pdf]

Table S2. Predicted discontinuous epitope(s) of Cov19B

No.	Residues	Number of residues	Score
1	A:D53, A:N54, A:K55	3	0.986
2	A:N397, A:S398, A:V399, A:A400, A:Y401, A:S402, A:N403, A:N404, A:K405, A:K406, A:E407, A:Q408, A:D409, A:K410, A:N411, A:T412, A:K413, A:K414, A:A552, A:S553, A:I554, A:E555, A:K556, A:S557, A:N558, A:I559, A:A560, A:A561, A:Y562, A:T563, A:L564, A:D565, A:S566, A:K567, A:T568, A:Q569, A:S570, A:L571, A:A572, A:A573, A:Y574, A:L575, A:P576, A:I577	44	0.855
3	A:M1, A:S2, A:Q3, A:C4, A:V5, A:N6, A:F7, A:R8, A:T9, A:R10, A:T11, A:Q12, A:L13, A:P14, A:S15, A:A16, A:Y17, A:K27, A:K28, A:L29, A:P30, A:F31, A:S33, A:N34, A:V35, A:T36, A:W37, A:F38, A:H39, A:A40, A:I41, A:H42, A:V43, A:S44, A:G45, A:T46, A:N47, A:G48, A:T49, A:K50, A:R51, A:K56, A:Y57, A:P58, A:F59, A:L60, A:D61, A:V62, A:Y63, A:H64, A:H65, A:K66, A:N67, A:N68, A:K69, A:S70, A:W71, A:M72, A:E73, A:K74, A:K75, A:M76, A:D77, A:L78, A:E79, A:G80, A:K81, A:F107, A:S108	69	0.763
4	A:P148, A:N149, A:I150, A:T151, A:N152, A:L153, A:C154, A:P155, A:F156, A:D157, A:E158, A:V159, A:F160, A:N161, A:A162, A:T163, A:K164, A:F165, A:A166, A:S167, A:V168, A:Y169, A:A170, A:W171, A:N172, A:K174, A:R175, A:I176, A:S177, A:N178, A:C179, A:V180, A:A191, A:T208, A:N209, A:Y211, A:A212, A:D213, A:S214, A:F215, A:K216, A:K217, A:G218, A:N219,	113	0.727

	A:E220, A:V221, A:S222, A:Q223, A:M224, A:A225, A:P226, A:G227, A:Q228, A:T229, A:G230, A:N231, A:I232, A:A233, A:D234, A:Y235, A:N236, A:Y237, A:K238, A:L239, A:P240, A:G249, A:S250, A:N251, A:Y252, A:N253, A:Y254, A:R255, A:Y256, A:R257, A:L258, A:F259, A:R260, A:K261, A:S262, A:N263, A:L264, A:K265, A:P266, A:F267, A:E268, A:R269, A:D270, A:I271, A:S272, A:T273, A:E274, A:I275, A:Y276, A:Q277, A:A278, A:G279, A:N280, A:K281, A:P282, A:C283, A:N284, A:G285, A:V286, A:A287, A:G288, A:V289, A:N290, A:C291, A:Y292, A:F293, A:P294, A:L295, A:R296		
5	A:R471, A:Y474, A:E475, A:P476, A:Q477, A:I478, A:I479, A:T480, A:T481, A:H482, A:K483, A:K484, A:V485, A:N486, A:N487, A:T488, A:V489, A:Y490, A:D491, A:P492, A:L493, A:Q494, A:P495, A:E496, A:L497, A:E498, A:S499, A:D517, A:L518, A:G519, A:D520, A:I521, A:S522, A:G523, A:I524, A:K525, A:K526, A:S527, A:C528, A:C529, A:K530, A:F531, A:D532, A:E533, A:L540, A:K541, A:G542, A:V543, A:K544, A:L545, A:H546, A:Y547, A:T548, A:K549, A:K550, A:F551	56	0.657
6	A:T303, A:K306, A:A307, A:P308, A:A309, A:T310, A:V311, A:C312, A:G313, A:P314, A:K315, A:K322, A:N323, A:K324, A:K325, A:E326, A:S327, A:N328, A:K329, A:K330, A:F331, A:L332, A:P333	23	0.622
7	A:R464, A:V465, A:F467, A:C468, A:K469, A:N472, A:F473	7	0.613
8	A:R23, A:G24, A:V25, A:Y26	4	0.613
9	A:Y360, A:V361, A:N362, A:N363, A:S364, A:Y365, A:E366, A:T374, A:Y389, A:T390, A:M391, A:S392, A:L393, A:G394, A:V395, A:E396	16	0.563

10	A:K183, A:N185, A:F186, A:A187, A:H188, A:F189	6	0.536
11	A:P435, A:S436, A:S439, A:K440	4	0.532
