# Supplementary material for: Designing a novel vaccine against COVID-19 based on spike SARS-Cov-2 notable mutations using immunoinformatics approaches
Source: PLoS One. 2026 Feb 26;21(2):e0334662. doi: 10.1371/journal.pone.0334662 (PMC12944808; doi:10.1371/journal.pone.0334662)
Supplement: S3 Table — (PDF) [file pone.0334662.s003.pdf]

Table S3. Predicted discontinuous epitope of Cov19T

No.	Residues	Number of residues	Score
1	A:D620, A:I621, A:A622, A:D623, A:T624, A:A625, A:D626, A:A627, A:V628, A:A629, A:A630, A:Y631, A:Y632, A:Q633, A:G634, A:V635, A:N636, A:T637, A:E638, A:V639, A:P640, A:V641, A:A642, A:A643, A:Y644, A:V645, A:F646, A:Q647, A:T648, A:R649, A:A650, A:G651, A:C652, A:L653, A:I654, A:A655, A:A656, A:Y657, A:L658, A:L659, A:L660, A:G663	42	0.839
2	A:L937, A:S938, A:C939, A:L940, A:P941, A:K942, A:E943, A:E944, A:Q945, A:I946, A:G947, A:K948, A:C949, A:S950, A:T951, A:R952, A:G953, A:R954, A:K955, A:C956, A:C957, A:R958, A:R959, A:K960, A:E962, A:A963, A:A964, A:A965, A:K966, A:A967, A:K968, A:F969, A:V970, A:A971, A:A972, A:W973, A:T974, A:L975, A:K976, A:A977, A:A978, A:A979, A:E980, A:A981, A:A982, A:A983, A:K984	47	0.809
3	A:E864, A:K865, A:N866, A:F867, A:G868, A:P869, A:G870, A:P871, A:G872, A:Y873, A:E874, A:Q875, A:Y876, A:I877, A:K878, A:P880, A:W881	17	0.701
4	A:V516, A:D517, A:L518, A:G519, A:D520, A:I521, A:S522, A:G523, A:I524, A:K525, A:K526, A:S527, A:C528, A:C529, A:K530, A:F531, A:D532, A:E533, A:D534, A:D535, A:S536, A:E537, A:K541, A:G542	24	0.686

5	A:P911, A:V912, A:P913, A:G914, A:H915, A:G916, A:G917, A:I918, A:I919, A:N920, A:T921, A:L922, A:Q923, A:K924, A:Y925, A:Y926, A:C927, A:R928, A:V929, A:R930, A:G931	21	0.665
6	A:A552, A:S553, A:I554, A:E555, A:K556, A:S557, A:N558, A:A560, A:A561, A:Y562, A:T563, A:L564, A:G760, A:G761, A:V762, A:S763, A:V764, A:I765, A:T766, A:P767, A:G768, A:P769, A:G770, A:P771, A:G772, A:G773, A:A774, A:G775, A:I776, A:C777, A:A778, A:S779, A:Y780, A:Q781, A:T782, A:Q783, A:G784, A:P785, A:G786, A:P787, A:G788, A:P789, A:I790, A:N791, A:F792, A:T793, A:I794, A:S795, A:V796, A:T797, A:T798, A:E799, A:I800, A:P802, A:T813, A:S814, A:V815, A:D816, A:C817, A:T818, A:M819, A:Y820, A:G821, A:P822, A:G823, A:P824, A:G825, A:A826, A:L827, A:Q828, A:I829, A:P830, A:F831, A:A832, A:M833, A:Q834, A:Y837	77	0.627
7	A:Y677, A:I678, A:A679, A:I680, A:V681, A:M682, A:V683, A:T684, A:I685, A:M686, A:A687, A:A688, A:F690, A:V691, A:L692, A:L693, A:P694, A:L695, A:V696, A:S697, A:G698, A:P699, A:G700, A:P701	24	0.583
8	A:G741, A:P742, A:G743, A:P744, A:G745, A:P746, A:Q747	7	0.535
