# Supplementary material for: Designing a novel vaccine against COVID-19 based on spike SARS-Cov-2 notable mutations using immunoinformatics approaches
Source: PLoS One. 2026 Feb 26;21(2):e0334662. doi: 10.1371/journal.pone.0334662 (PMC12944808; doi:10.1371/journal.pone.0334662)
Supplement: S4 Table — (PDF) [file pone.0334662.s004.pdf]

Table S4. Summary of the top 10 models for Cov19B-Heavy chain

**Rank**	**1**	**2**	**3**	**4**	**5**	**6**	**7**	**8**	**9**	**10**
**Docking Score**	-332.07	-323.77	-319.82	-315.52	-312.98	-305.20	-302.96	-296.53	-289.86	-289.31
**Confidence Score**	0.9745	0.9700	0.9676	0.9648	0.9630	0.9571	0.9552	0.9493	0.9425	0.9419
**Ligand rmsd (Å)**	138.65	142.74	158.30	128.29	153.31	149.58	145.26	157.18	155.92	129.88
**Interface residues (model)**	1	2	3	4	5	6	7	8	9	10
