# Supplementary material for: Designing a novel vaccine against COVID-19 based on spike SARS-Cov-2 notable mutations using immunoinformatics approaches
Source: PLoS One. 2026 Feb 26;21(2):e0334662. doi: 10.1371/journal.pone.0334662 (PMC12944808; doi:10.1371/journal.pone.0334662)
Supplement: S5 Table — (PDF) [file pone.0334662.s005.pdf]

Table S5. Summary of the top 10 models for Cov19B-Light chain

**Rank**	**1**	**2**	**3**	**4**	**5**	**6**	**7**	**8**	**9**	**10**
**Docking Score**	-323.14	-300.16	-298.60	-295.40	-289.43	-285.99	-285.23	-282.02	-279.15	-278.02
**Confidence Score**	0.9696	0.9527	0.9513	0.9482	0.9421	0.9382	0.9373	0.9334	0.9298	0.9283
**Ligand rmsd (Å)**	178.89	173.73	173.98	183.28	181.92	214.02	187.24	210.31	186.59	174.54
**Interface residues (model)**	1	2	3	4	5	6	7	8	9	10
