# Supplementary material for: Designing a novel vaccine against COVID-19 based on spike SARS-Cov-2 notable mutations using immunoinformatics approaches
Source: PLoS One. 2026 Feb 26;21(2):e0334662. doi: 10.1371/journal.pone.0334662 (PMC12944808; doi:10.1371/journal.pone.0334662)
Supplement: S6 Table — (PDF) [file pone.0334662.s006.pdf]

Table S6. Summary of the top 10 models for Cov19T-Heavy chain

**Rank**	**1**	**2**	**3**	**4**	**5**	**6**	**7**	**8**	**9**	**10**
**Docking Score**	-328.47	-326.41	-325.26	-318.48	-312.32	-305.71	-296.94	-293.66	-291.70	-289.90
**Confidence Score**	0.9726	0.9715	0.9708	0.9667	0.9625	0.9575	0.9497	0.9465	0.9445	0.9426
**Ligand rmsd (Å)**	224.26	227.82	215.93	211.86	214.21	254.58	210.27	224.21	252.62	216.51
**Interface residues (model)**	1	2	3	4	5	6	7	8	9	10
