# Supplementary material for: Designing a novel vaccine against COVID-19 based on spike SARS-Cov-2 notable mutations using immunoinformatics approaches
Source: PLoS One. 2026 Feb 26;21(2):e0334662. doi: 10.1371/journal.pone.0334662 (PMC12944808; doi:10.1371/journal.pone.0334662)
Supplement: S7 Table — (PDF) [file pone.0334662.s007.pdf]

Table S7. Summary of the top 10 models for Cov19T-Light chain

**Rank**	**1**	**2**	**3**	**4**	**5**	**6**	**7**	**8**	**9**	**10**
**Docking Score**	-310.24	-295.89	-273.28	-273.12	-271.81	-269.97	-268.30	-267.00	-266.49	-266.47
**Confidence Score**	0.9610	0.9487	0.9217	0.9215	0.9195	0.9168	0.9142	0.9121	0.9113	0.9113
**Ligand rmsd (Å)**	182.78	188.30	205.81	217.74	195.00	183.89	195.55	215.06	183.72	200.91
**Interface residues (model)**	1	2	3	4	5	6	7	8	9	10
