# Supplementary figures and images for: Designing a novel vaccine against COVID-19 based on spike SARS-Cov-2 notable mutations using immunoinformatics approaches
Source: PLoS One. 2026 Feb 26;21(2):e0334662. doi: 10.1371/journal.pone.0334662 (PMC12944808; doi:10.1371/journal.pone.0334662)

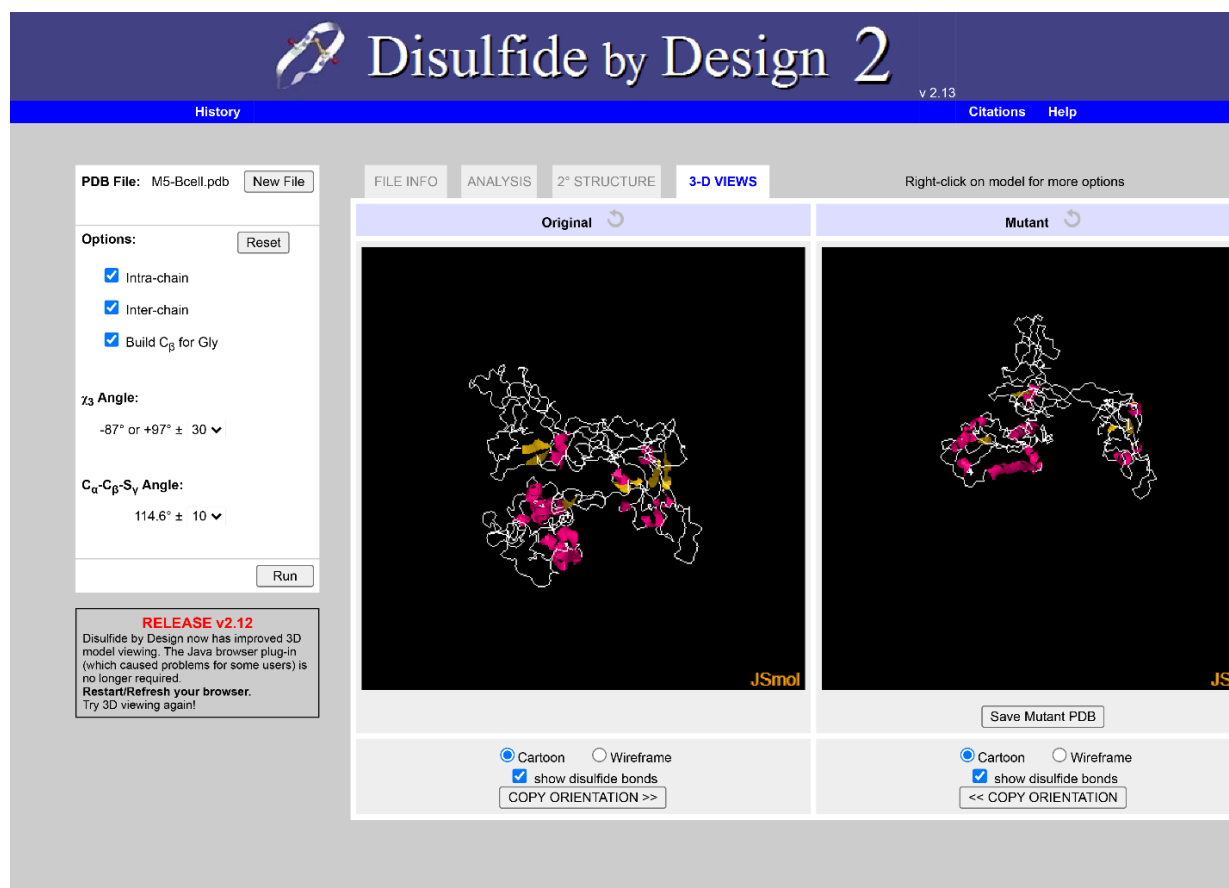

**Figure S1.** Results of Engineering disulfide for Cov19-B 3D structure

Supplement: S1 Fig — (PDF) [file pone.0334662.s008.pdf]

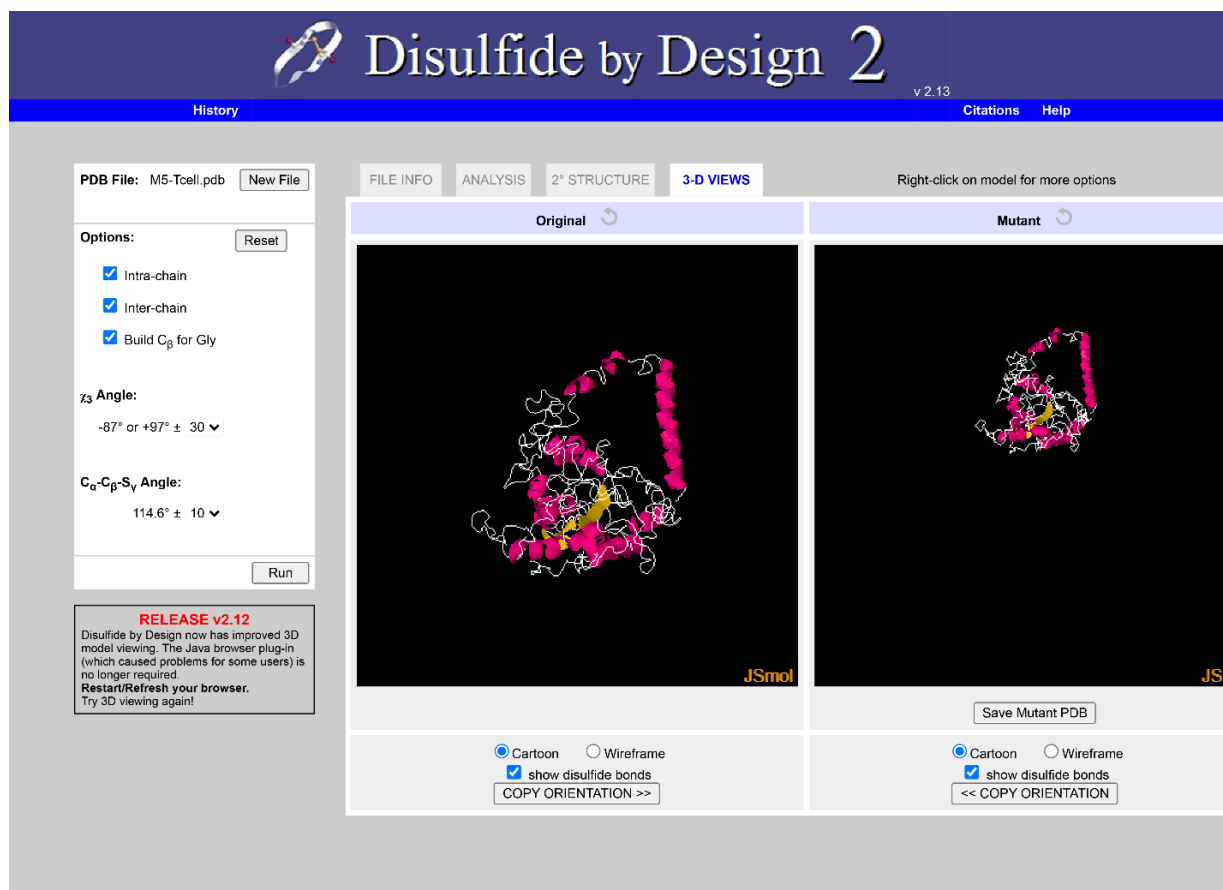

**Figure S2.** Results of Engineering disulfide for Cov19-T 3D structure

Supplement: S2 Fig — (PDF) [file pone.0334662.s009.pdf]
